# γ-Aminobutyrate (GABA) Regulated Plant Defense: Mechanisms and Opportunities

**DOI:** 10.3390/plants10091939

**Published:** 2021-09-17

**Authors:** Barry J. Shelp, Morteza Soleimani Aghdam, Edward J. Flaherty

**Affiliations:** 1 Department of Plant Agriculture, University of Guelph, Guelph, ON N1G 2W1, Canada; eflahert@uoguelph.ca; 2 Department of Horticultural Science, Imam Khomeini International University, Qazvin 34148-96818, Iran; soleimaniaghdam@eng.ikiu.ac.ir

**Keywords:** abiotic stress, antioxidants, biostimulants, biotic stress, GABA, metabolism, phytohormones, reactive oxygen species, signaling, tricarboxylic acid cycle

## Abstract

Global climate change and associated adverse abiotic and biotic stress conditions affect plant growth and development, and agricultural sustainability in general. Abiotic and biotic stresses reduce respiration and associated energy generation in mitochondria, resulting in the elevated production of reactive oxygen species (ROS), which are employed to transmit cellular signaling information in response to the changing conditions. Excessive ROS accumulation can contribute to cell damage and death. Production of the non-protein amino acid γ-aminobutyrate (GABA) is also stimulated, resulting in partial restoration of respiratory processes and energy production. Accumulated GABA can bind directly to the aluminum-activated malate transporter and the guard cell outward rectifying K^+^ channel, thereby improving drought and hypoxia tolerance, respectively. Genetic manipulation of GABA metabolism and receptors, respectively, reveal positive relationships between GABA levels and abiotic/biotic stress tolerance, and between malate efflux from the root and heavy metal tolerance. The application of exogenous GABA is associated with lower ROS levels, enhanced membrane stability, changes in the levels of non-enzymatic and enzymatic antioxidants, and crosstalk among phytohormones. Exogenous GABA may be an effective and sustainable tolerance strategy against multiple stresses under field conditions.

## 1. Introduction

The world population is predicted to be 9–10 billion people by 2050, so that a 60^−1^10% increase in global food production will be required as more marginal lands are being used for agricultural purposes [1]. Furthermore, at the current rate of global warming, the temperature is projected to increase by 1.5−2.4 °C [2]. With a 1.5 °C increase, heavy precipitation and associated flooding will intensify and be more frequent in most regions of Africa, Asia, North America and Europe. Additionally, more frequent and/or severe droughts will occur in a few regions on all continents except Asia. With further global warming, every region is projected to increasingly experience concurrent and multiple climatic changes (e.g., salinity, O_2_ deprivation, acidity, heavy metals), which will adversely affect plant growth and development. These changing climatic conditions could facilitate the geographic expansion and aggressiveness of phytopathogens and modify host susceptibility [3]. Therefore, it is imperative to develop crop production systems that are more sustainable under stress conditions [4].

Under extreme environments, the overaccumulation of oxygen radicals and their derivatives (e.g., superoxide anion, O_2_^•−^, hydroxyl radical, ^•^OH; singlet oxygen, ^1^O_2_; hydrogen peroxide, H_2_O_2_), known as reactive oxygen species (ROS), can lead to cellular damage, programmed cell death and lower plant productivity [5]. ROS are formed in many plant cell compartments, including chloroplasts, mitochondria, peroxisomes and plasma membrane. An optimum level of ROS is generally maintained by antioxidant defenses, so that a signal is transmitted to the nucleus through redox reactions, using the mitogen-activated protein kinase pathway in a variety of cellular mechanisms, to increase tolerance against diverse abiotic stresses [6].

There is considerable interest in improving stress tolerance using breeding and genetic engineering approaches, and exogenous application of natural compounds, including primary and secondary metabolites [4]. γ-Aminobutyrate (GABA) is a ubiquitous four-carbon, non-proteinogenic amino acid, which functions as both metabolite and signal in response to abiotic and biotic stresses [7,8,9,10,11,12,13,14,15,16,17,18,19,20,21,22,23]. The GABA shunt involves the activity of evolutionary-conserved enzymes that bypass two steps of the mitochondrial tricarboxylic acid cycle (TCAC), partially restore the stress-induced changes in respiratory processes, and alleviate oxidative injury. In addition, GABA accumulated during stress can bind directly to the aluminum-activated malate transporter (ALMT) and the guard cell outward rectifying K^+^ (GORK) channel, thereby improving stress tolerance. Under acidic conditions, heavy metals activate malate efflux via root ALMT, resulting in heavy metal-malate complexes that are not readily absorbed [13].

Here, we review the pathways for, and the compartmentation of, GABA metabolism in plants, identifying some key gaps in our knowledge of the mechanisms involved, and then discuss stress-induced changes in flux, energy generation, redox balance and ROS. Second, we review evidence for GABA signaling in roots and stomata, and then discuss how the accumulation of GABA can influence K^+^ and malate efflux, resulting in hypoxia, drought or aluminum tolerance. Third, we discuss the improvement of biotic stress resistance by the genetic enhancement of endogenous GABA. Finally, we discuss the improvement of abiotic stress tolerance using exogenous GABA to promote GABA, antioxidant, phytohormone and secondary pathways. These findings suggest that GABA application could be an appropriate treatment for dealing with different or simultaneous stresses in a field setting.

## 2. GABA Metabolism and Its Response to Abiotic Stress

### 2.1. Pathways and Compartmentation

**Figure 1:**
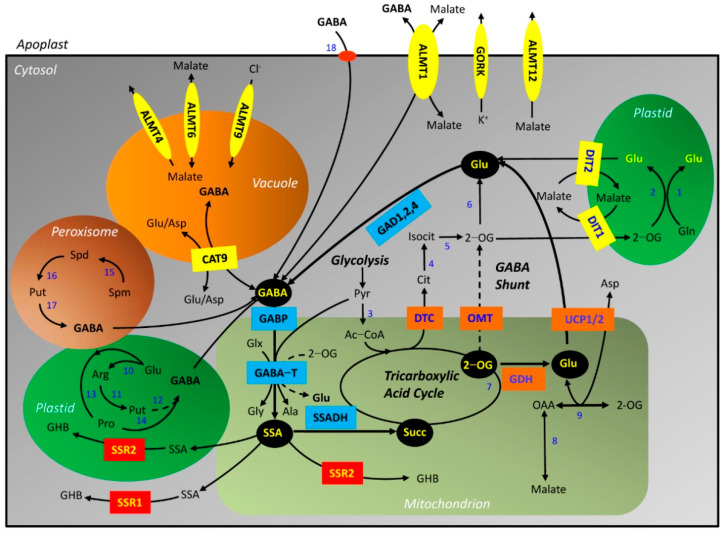
Model for stress-mediated GABA metabolism and signaling in *Arabidopsis*. The black ovals and blue squares represent important metabolites and enzymes/transporters, respectively, in the GABA shunt. Steps lacking convincing experimental support are shown as dashed lines. The orange squares represent enzymes/transporters that potentially link the tricarboxylic acid cycle back to the GABA shunt, the yellow squares represent transporters that link the GABA shunt to other pathways and organelles, and the red squares represent enzymes catalyzing the detoxification of SSA. The yellow ovals represent recently−identified transporters that are potentially regulated by GABA. Abbreviations: Ac−CoA, acetyl−CoA; Ala, alanine; ALMT, aluminum−activated malate transporter; Arg, arginine; Asp, aspartate; CAT, cationic amino acid transporter; Cit, citrate; DIT, dicarboxylate translocator; DTC, dicarboxylate/tricarboxylate carrier; GABA, γ−aminobutyrate; GABA−T, GABA transaminase; GABP, GABA permease; GAD, glutamate decarboxylase; GDH, glutamate dehydrogenase; GHB, γ−hydroxybutyrate; Gln, glutamine; Glu, glutamate; SSR, succinic semialdehyde reductase; Isocit, isocitrate; OAA, oxaloacetate; 2−OG, 2−oxoglutarate; OMT, 2−oxoglutarate/malate translocator; Pro, proline; Put, putrescine; Pyr, pyruvate; Spd, spermidine; Spm, spermine; Succ, succinate; SSA, succinic semialdehyde; SSADH; succinic semialdehyde dehydrogenase; UCP, uncoupling protein. Additional enzymes are indicated as numbers: 1, glutamine synthetase; 2, ferredoxin−dependent glutamate synthase; 3, pyruvate dehydrogenase complex; 4, aconitase; 5, isocitrate dehydrogenase; 6, glutamate:oxaloacetate (aspartate) transaminase or glutamate:pyruvate (alanine) transaminase; 7, 2−oxoglutarate dehydrogenase and succinyl−CoA ligase; 8, malate dehydrogenase; 9, aspartate transaminase; 10, urea cycle; 11, arginine decarboxylase, agmatine iminohydrolase and N−carbamoylputrescine amidohydrolase; 12, copper amine oxidase and aldehyde dehydrogenase 10A8; 13, ∆^1−^pyrroline-5−carboxylate synthetase and ∆^1−^ pyrolline-5−carboxylate reductase; 14, spontaneous decarboxylation of proline to pyrrolidin^−1^−yl, which is easily converted to Δ^1−^pyrroline/4−aminobutanal and then GABA via aldehyde dehydrogenase (ALDH10A8); 15, polyamine oxidase (PAO2−4); 16, polyamine oxidase (PAO2,3); 17, uncertain copper amine oxidase and aldehyde dehydrogenase (ALDH10A9); 18, proline transporter (PROT1,2,3) or GABA transporter (GAT1) (modified from [17]). Important *Arabidopsis thaliana* gene names and identifiers are given in Supplementary Table S1.

Figure 1 shows an updated model of the metabolic and signaling pathways for GABA in plants, emphasizing vegetative organs. The model is based primarily on research with *Arabidopsis thaliana* (L.) Heynh., but where necessary, supporting evidence from other model plant systems such as petunia (Petunia x hybrida Juss.), tobacco (*Nicotiana tabaccum* L.), soybean (*Glycine max* (L.) Merr.), tomato (*Solanum lycopersicum* L.), rice (*Oryza sativa* L.), wheat (*Triticum aestivum* L.) and corn (*Zea mays* L.) is mentioned in this article [8,13,14,16]. While the interaction of the GABA shunt (i.e., cytosolic glutamate (Glu) decarboxylase (GAD), mitochondrial GABA transaminase (GABA-T), and mitochondrial succinic semialdehyde (SSA) dehydrogenase (SSADH)) with the TCAC may be considered the central feature of this model, there is increasing evidence for the involvement of several branch pathways in the homeostasis of cellular GABA [17,18,19].

#### 2.1.1. Biosynthesis of GABA from Glutamate, Polyamines or Proline

GAD is the direct source of GABA in the cytosol [24,25,26,27,28] (Figure 1). It is irreversible, pyridoxal 5′-phosphate-dependent, specific for L-Glu (*K*_m_s for various plant GADs range from 3 to 32 mM), and maximally active at approximately pH 5.8 [15]. Many plant GADs possess a C-terminal domain that binds the Ca^2+^/calmodulin (CaM) complex, thereby activating GAD activity at neutral pH [15,29,30,31,32,33,34,35]. *Arabidopsis* has five GAD genes in total, but only AtGAD1,2,4 possess the C-terminal domain [15,16]. Thus, stress-induced increases in cytosolic Ca^2+^/CaM complexation or H^+^ can activate/stimulate GAD activity [9,36,37,38].

The GABA level in the roots of the *Arabidopsis atgad1* mutant is 15% of the wild-type (WT), but the Glu level increases [39]. The GABA level in the shoot of the *atgad2* mutant is less than 25% of that in the WT, but the level in the roots is unaffected [40]. In contrast, the GABA level in the *atgad4* mutant is unaffected, compared to the WT [41]. Transgenic tobacco plants overexpressing a mutant petunia GAD lacking the autoinhibitory domain (*GAD*∆*C*) exhibit severe morphological abnormalities, such as short stems, with high GABA and low Glu levels (18 and 8 mol% of total free amino acids, respectively, vs. 9 and 38% in the WT), as well as flowers that do not form pollen and abscise prematurely [29]. Notably, McLean et al. [42] have identified transgenic tobacco plants overexpressing *NtGAD*∆*C* and exhibiting a normal phenotype, while the GABA levels are approximately 3-fold higher than in the WT.

Cytosolic GABA may also be derived indirectly from the metabolism of polyamines (PA) in organelles [43,44] (Figure 1). In *Arabidopsis*, the primary PA, putrescine (Put), is generated from the secondary PA, spermidine (Spd), and the tertiary PA, spermine (Spm), via FAD-dependent PA oxidases (PAO) in the peroxisome [45]. Put is produced in the plastid from arginine via arginine decarboxylase, agmatine imidohydrolase and carbamoylputrescine amidohydrolase [17,44]. 4-Aminobutanal (ABAL) can be derived in the peroxisome from both Spd and Put via two copper-dependent diamine oxidase activities (AtCuAOα3 and AtCuAOζ based on the terminology in Tavladoraki et al. [46]) [47]. Terminal oxidation of Put in the plastid requires a plastidial CuAO, likely with a preference for diamines as substrates [17].

Early support for the existence of ABAL/pyrroline dehydrogenase in plants is available from the production of radiolabeled GABA from exogenously supplied radiolabeled Put, and the suppression of GABA production by the addition of aminoguanidine, a CuAO inhibitor [44,48]. More recent research demonstrated the conversion of ABAL into GABA via ABAL/pyrroline dehydrogenase activities (plastidial AtALDH10A8 and peroxisomal AtALDH10A9) [49,50]. Both AtALDH10A8 and AtALDH10A9 have strong alkaline pH optima, are NAD-dependent and use ABAL, as well as 3-aminopropanal and γ-trimethylaminobutyraldehyde (all with *K*_m_s in the low micromolar range) as substrates to produce GABA, β-alanine and γ-butyrobetaine, respectively [50,51]. They are also prone to substrate inhibition. *ataldh10A8,9* seedlings are phenotypically normal, and the GABA and Glu levels are similar across the mutants and WT [50].

Proline is another potential indirect source of cytosolic GABA [52]. It is derived in the plastid/cytosol from Glu via NADP-dependent ∆^1^-pyrroline-5-carboxylate synthetase and NADP-dependent ∆^1^-pyrroline-5-carboxylate reductase [53]. Proline reacts with a hydroxyl radical, resulting in H–abstraction from the amine group, then spontaneous decarboxylation of proline and formation of pyrrolidin-1-yl [52]. Pyrrolidin-1-yl can easily be converted to Δ^1^–pyrroline/ABAL and oxidized to GABA via ABAL/pyrroline dehydrogenase activity. To date, there is no direct evidence for the contribution of proline to GABA production in planta.

#### 2.1.2. Conversion of GABA to Succinate or γ-Hydroxybutyrate

The mitochondrial-localized bidirectional amino acid transporter/GABA permease (AtBAT1/GABP) links the anabolic and catabolic portions of the GABA shunt [54] (Figure 1). However, GABA uptake by mitochondria isolated from the *atgabp* mutant is not eliminated, suggesting the possible existence of other mitochondrial GABA carriers with overlapping or redundant functions [16]. The substrate preference for AtGABP requires clarification (arginine, Glu and lysine, but not GABA and proline [55]; GABA, but not proline [54]; *K*_m_ Spd = 55 μM, *K*_m_ Put = 85 μM, *K*_m_ arginine = 1.4 mM [56]).

The pyruvate- and glyoxylate-dependent GABA-T (GABA-TP) catalyzes conversion of GABA to SSA in the mitochondrion [57,58,59] (Figure 1). It is reversible with a pH optimum of 9 and *K*_m_s for GABA, pyruvate and glyoxylate of 0.18−0.34 mM, 0.14 mM and 0.11 mM, respectively [57,58]. This enzyme is often portrayed as possessing 2-oxoglutarate-dependent activity (GABA-TOG) [60,61,62]; however, an *AtGABA-TOG* gene has not yet been identified. In our opinion, a recent paper describing sugarcane GABA-TOG activity lacks rigor [63], and the existence of a plant GABA-TOG remains an open question [15]. Furthermore, the detection of GABA-TOG activity in crude extracts should be treated with skepticism [64,65,66]. The *atgaba-tp* mutant is phenotypically normal, except for lower seed production, and the leaf GABA level can increase up to 16-fold without an effect on the Glu level [58,67,68,69,70].

The conversion of SSA into succinate in the mitochondrion is catalyzed by SSADH activity [71,72] (Figure 1). The AtSSADH is reversible with a pH optimum of 9–9.5 and feedback-regulation by NADH and ATP (*K*_m_ SSA = 15 μM, *K*_m_ NAD = 130 μM, *K*_i_ NADH = 122 μM, *K*_i_ ATP = 8 mM). The *atssadh* mutant overaccumulates GABA (28 nmol g^−1^ FM) and H_2_O_2_ by 2- and 4-fold, respectively [73,74]. Succinate can contribute to the production of C skeletons and NADH via the TCAC and the generation of ATP via the mitochondrial electron transport chain (mETC), which in turn, prevents the accumulation of ROS [25,28,74]. Notably, SSADH and nearly every enzyme involved in the TCAC and mETC are succinylated, but the supply of succinate is limited during oxidative stress by lower OGDH and succinyl-CoA ligase activities [75,76]. These findings support the hypothesis that GABA shunt activity is necessary for the modification and regulation of respiratory activities to ensure an adequate ATP supply and minimize the generation of ROS (73).

SSA can be reduced to γ-hydroxybutyrate (GHB) via NADPH-dependent glyoxylate/SSA reductases in the mitochondrion/plastid (AtGLYR2/SSR2) and cytosol (AtGLYR1/SSR1) [77,78,79,80,81,82,83] (Figure 1). These proteins have affinity for SSA in the low millimolar range, glyoxylate in the low micromolar range, and NADPH in the low micromolar range, and are competitively inhibited by NADP^+^ (*K*_i_ = 1–3 μM) [78,82,83,84,85]. The *atglyr1,2* mutants and *NAD kinase 1* overexpression line accumulate less and more GHB, respectively, with submergence than the WT [86]. The growth of plantlets or roots of various *Arabidopsis* lines with altered GLYR activity responds differentially to SSA or glyoxylate under chilling conditions [83]. Together, these findings are consistent with an elevated rate of SSA conversion to GHB with cold and low O_2_, and suggest that AtGLYR1,2 are part of an adaptive response to stress-induced changes in redox balance [83]. Notably, the rice *osglyr1/2* double mutant displays stunted growth under photorespiratory conditions, compared to the WT [87], validating our earlier hypothesis that the GLYRs reduce both glyoxylate and SSA in planta [88]. The two GLYRs function in a redundant manner, which would be consistent with the diffusion of SSA and glyoxylate from their sites of origin. The fate of GHB in plants is uncertain; however, it could be linked to acetyl-CoA and fatty acid metabolism [12,16].

#### 2.1.3. Biosynthesis of Glutamate from Succinate and 2-Oxoglutarate

Succinate is converted to citrate and 2-OG in the TCAC [25,28] (Figure 1). Two potential routes have been proposed for diverting these two important TCAC intermediates to the generation of cytosolic Glu [19]. The first involves the export of citrate from the mitochondrion via the dicarboxylate/tricarboxylate carrier (AtDTC) and its conversion to isocitrate and then 2-OG in the cytosol. The second involves the export of 2-OG via a 2-OG/malate translocator (AtOMT) [19]; contrary to a recent suggestion [89], we could find no evidence in the literature for a mitochondrial-specific OMT. In both cases, 2-OG would be converted to Glu via cytosolic transaminase activities. A third route is also possible, involving the direct synthesis of Glu from 2-OG via the mitochondrial glutamate dehydrogenase (GDH) and then export via the uncoupling proteins AtUCP1,2 [90,91]. The UCP is known to decrease the electrochemical gradient across the mitochondrial inner membrane, and prevent the over-reduction of the mETC [92].

Glu availability is an essential regulator of GAD activity, and the generation of cytosolic Glu from succinate bypasses two reactions of the TCAC (i.e., 2-OGDH and succinyl-CoA ligase). However, it does not exclude participation in the cytosolic GAD reaction of Glu originating in the plastid via the glutamine synthetase/glutamate synthase (GS/GOGAT) cycle [93] (Figure 1). The movement of 2-OG and Glu across the plastidial inner membrane is mediated by dicarboxylate translocators (AtDIT) [94].

#### 2.1.4. GABA Transport

*Arabidopsis* grows efficiently on GABA as the sole N source, providing the first strong evidence for GABA uptake by plant cells [95] (Figure 1). Two types of plasma membrane-located amino acid transporters, amino acid permease 3 (AtAAP3) and proline transporter 1,2,3 (AtPROT1,2,3), transport GABA (*K*_m_ = 12.0 and 1.7–5 mM, respectively [96,97]). AtPROT2- and SlPROT1-mediated GABA transport is inhibited by proline and quaternary ammonium compounds [95,97]. AtGAT1 is a high-affinity plasma membrane-localized, proton-coupled transporter that is apparently specific for GABA (not transporting Glu or Asp) (*K*_m_ = 10 μM) [98,99]. Endogenous GABA in the *atgat1* mutant is unaffected by the addition of exogenous GABA, but it increases in the WT, confirming that AtGAT1 plays a role in GABA influx into the cell [99].

GABA is released from asparagus mesophyll cells via an unknown mechanism [100], but evidence is emerging for the bi-directional transport of GABA across the plasma membrane via the wheat root, aluminum-activated malate transporter (TaALMT1) [18,101,102] (Figure 1). AtALMT1 is highly homologous to TaALMT1, and there are plant ALMTs that encode channels with a preference for malate, chloride or nitrate [14]. However, their capacity to transport GABA has not yet been investigated.

To date, two organellar transporters for GABA have been described. The mitochondrial AtGABP was the first (see Section 2.1.2). Another one, SlCAT9, is localized to the tonoplast and links the cytosol with the vacuolar compartment, operating strictly in a stoichiometric exchange mode with Glu and aspartate so that the osmolarity of the vacuole does not change [103] (Figure 1).

### 2.2. Precursor–Product Relations and Flux in the GABA Shunt

Kaplan et al. [104] showed that exposure to 4 °C results in the sharp accumulation of both GABA and succinate from 12 to 24 h in the aerial portion of *Arabidopsis* plants. Subsequently, GABA declines, but succinate remains steady for 72 h. GHB accumulates sharply from 24 to 48 h and then declines to 96 h. Glu accumulates in a linear fashion from 12 to 96 h, whereas Put does so from 24 h. Furthermore, Espinoza et al. [105] demonstrated that exposure to 4 °C increases the GABA and alanine levels from 2 to 30 h; the GABA level remains steady for the remaining 28 h. Proline and Put begin to accumulate shortly thereafter (from 18–22 to 58 h), whereas the Spd level does not change. Interestingly, the succinate and malate levels decrease early in the time course and then remain steady. In contrast, the levels of 2-OG and glutamine (Gln) initially decline and then increase.

Some generalizations are possible from these two studies: (i) the cold-induced accumulation of GABA, alanine, succinate and GHB are not correlated with the availability of Put and proline; (ii) succinate and GHB may be concomitantly generated from SSA; (iii) SSA and succinate turnover may be restricted or an alternate source of succinate exists; and, (iv) Glu/Gln metabolism is altered under cold conditions. A definitive explanation for the increasing accumulation of Glu is not possible, despite the accumulation of three well-known products of Glu metabolism (i.e., GABA, Put and proline), though protein hydrolysis is known to be stimulated by low temperature [106].

Treatment with 50–150 mM NaCl for 6 d increases the GABA level in soybean (*Glycine max* (L.) Merr.) roots by 11- to 17-fold, compared to the control, as well as the diamine oxidase activity by 52–86%, but decreases the levels of Put, Spd and Spm [48]. Aminoguanidine inhibition of diamine oxidase activity increases the Put level from 28 to 51 nmol g^−1^ fresh mass (FM), but decreases the GABA level from 10.8 to 6.6 μmol g^−1^ FM. While these findings could be interpreted as support for the derivation of GABA from Put [48], the molar stoichiometry (∆GABA/∆Put) deviates markedly from the 1:1 ratio expected if Put is a major source of GABA, suggesting that aminoguanidine also interferes with the generation of GABA from Glu.

Other studies have modeled the stress-induced changes in flux through the GABA shunt using suspension cultures. For example, the mean GABA, alanine, proline, Glu and Gln pools in control tomato (*Solanum lycopersicum* L.) cells are 1.2, 2.0, 0.12, 0.84 and 6.77 μmol g^−1^ FM, respectively, over a 2-d period, whereas they are 13.0, 6.26, 31.2, 1.22 and 2.71 μmol g^−1^ FM, respectively, in cells adapted to water stress (25% polyethylene glycol 6000) [107]. Computer simulation of ^15^N-labeling kinetics reveals that adaptation to water stress increases the N flux into GABA and alanine, suggesting high pyruvate availability and rapid turnover of both amino acids. The rate of GABA synthesis and catabolism, respectively, are 0.80 and 0.785 μmol h^−1^ g^−1^ FM in control cells, and 2.4 and 1.26 μmol h^−1^ g^−1^ FM in adapted cells. About 76% of the GABA is located in a metabolically inactive pool in unadapted cells, but only 38% in adapted cells. The proline pool increases by 300-fold due to greater synthetic rates from Glu and restricted oxidation, and the metabolically inactive Gln storage pool becomes depleted. Notably, the rate of nitrogen assimilation doubles, even though the total soluble protein (on a dry mass basis) decreases by 30%.

In related research, the mean GABA, alanine, proline, Glu and Gln pools in unadapted cowpea (*Vigna unguiculata* (L.) Walp) cells at 26 °C are 0.27, 5.37, 0.42, 1.79 and 4.11 μmol g^−1^ FM, respectively [108]. The GABA pool size in cells transferred to 42 °C increases to 1.85 and 3.24 μmol g^−1^ FM after 2 h and 1 d, respectively, and the other amino acids also accumulate, but less extensively than GABA. Total free amino acid levels increase approximately 1.5-fold after 1 d at 42 °C. The computer simulation suggests that heat shock induces a 63-fold increase in the rate of GABA synthesis over the first 2 h, without any change in the rate of GABA catabolism. The rate of GABA synthesis over the next 22 h increases 7-fold, and this is accompanied by a 3-fold increase in GABA catabolism. The rates of alanine and proline synthesis increase by 0.6 and 1.7-fold, respectively, over the 1-d period. The size of the free amino acid pool increases within 1 d, and the rate of protein synthesis decreases by 20–30%. An accelerated rate of protein degradation may also contribute to the effects of heat shock on the amino acid perturbations [108].

The stress-activated acceleration of flux through the GABA shunt can be explained by increases in GAD activity and GABA synthesis due to elevated levels of Glu (e.g., reduction in ATP availability, recycling of NH_3_ and protein synthesis; increase in protein degradation) [93,109,110,111], and the Ca^2+^/CaM complex or H^+^ in the cytosol. The GOGAT- or GDH-mediated regeneration of Glu from 2-OG in the plastid and mitochondrion, respectively, is sufficient to sustain the increased formation of cytosolic GABA for a limited duration. However, the relative importance of PAs and proline to GABA accumulation remains controversial [35].

### 2.3. Respiration, Redox Balance and Reactive Oxygen Species

Recent studies have assessed the interaction between GABA catabolism and respiration. For example, increases in GAD activity and GABA level (1-fold) in leaves of tomato plants after 5 d of exposure to 200 mM NaCl, together with the 25% decrease in succinate [112], suggest that the SSADH-mediated production of succinate is insufficient to sustain the average rate of respiration. An increase in H_2_O_2_ level supports this interpretation. Che-Othman et al. [89] also found a 1-fold increase in the GABA level in wheat seedlings 3 to 11 d after exposure to 150 mM NaCl, compared to the control. Furthermore, increases in GAD activity (pH 5.8), and the levels of succinate, 2-OG, Glu and Gln, together with decreases in the activity and abundance of pyruvate dehydrogenase and OGDH, and the levels of citrate, aconitate, fumarate and malate suggest that: (i) elevated GABA shunt activity accounts for an approximate 20% increase in the respiration rate of salt-stressed leaves, despite the lower potential for pyruvate oxidation by TCAC; and (ii) GOGAT and GDH are likely indicators of N assimilation into Glu [89].

Respiration in soybean roots is reduced by 40%, after 6 h of hypoxia, but the ATP and pyruvate supply is enhanced via an activated glycolytic pathway, and the cytosolic NAD^+^ is regenerated via fermentation reactions [113]. The direct flux of pyruvate into the TCAC is low, and the conversion of succinate to fumarate is markedly decreased due to restricted pyruvate dehydrogenase and succinate dehydrogenase activities. Pyruvate accumulation is minimized via the alanine transaminase- and GABA-TP-mediated formation of alanine, and the alanine transaminase reaction generates 2-OG for use by OGDH and succinyl CoA ligase to produce another ATP. The NAD^+^ required for oxidation of 2-OG is apparently produced by the anti-clockwise operation of the TCAC malate dehydrogenase, which increases malate accumulation. The carbon flux from SSA to succinate is not eliminated, even though the NADH/NAD^+^ ratio is presumably altered to some degree. GABA accumulates, at least in part, due to the stimulation of GAD activity by bound Ca^2+^/CaM or lower cytosolic pH. Overall, both GABA and succinate appear to be temporary storage metabolites that readily supply the TCAC when hypoxia is mitigated [114].

The hypoxia-induced increase in NADPH/NADP^+^ ratio might be attributed to increases in the activities of NAD kinases [86] and the oxidative pentose phosphate pathway [106]. In addition, there is evidence for NADPH-mediated reduction of alternative oxidases and then activation by either pyruvate or succinate [115], NADPH-mediated removal of SSA via SSR activity [78,83], and NADPH oxidase-mediated generation of H_2_O_2_ [116,117]. With the exception of hypoxia, abiotic stresses cause stomatal closure and decrease CO_2_ fixation, leading to the underutilization of NADPH, over-reduction of the photosynthetic electron transport chain, and the generation of ROS. Stomatal closure also increases Rubisco activity, leading to the glycolate oxidase-mediated generation of H_2_O_2_ [88].

### 2.4. Genetic Manipulation of Endogenous GABA Modifies the Abiotic Stress Phenotype

Table 1 describes examples wherein the phenotype of GABA pathway mutants is modified under stress conditions. In general, the *atgad2* single and *atgad1/2* double mutants of *Arabidopsis* do not accumulate GABA in response to salinity, drought or hypoxia, but they become hypersensitive to the stress [40,69,70,118]. This suggests that AtGAD4-, PA- or proline-derived GABA is insufficient to counter the stress under consideration, though *ataldh10A8,9* single mutants have been shown to decrease the GABA level and confer a hypersensitive salinity phenotype [50]. In contrast, the GABA level in the *atgaba-t* mutant increases with salinity or hypoxia and the phenotype is more tolerant to the stress [70,118]. Furthermore, the loss in GABA accumulation in the *atgad1/2* mutant results in more RBOHF/NADPH oxidase-mediated H_2_O_2_ production and K^+^ efflux via the GORK and Shaker-type outward rectifying K^+^ channels than in the *atgaba-t* mutant [70,117,118]. Surprisingly, another study reported that the GABA level in the shoot of the *atgaba-t* mutant doubles with salinity (28 μmol g^−1^ DM), while the succinate and 2-OG levels decrease or are unchanged, but the mutant becomes hypersensitive to salinity [68]. The *atssadh* mutant overaccumulates GABA, H_2_O_2_ and GHB in the absence of stress, and displays hypersensitivity to heat stress [73,74].

Knockdown of tomato *SlGAD1-4* prevents the salinity-induced GABA accumulation and confers a hypersensitive salinity phenotype, whereas knockdown of S*lGABA-T1-3* slightly increases the GABA level, while conferring a hypersensitive salinity phenotype [112] (Table 1). In contrast, knockdown of *SlSSADH* increases the GABA level and confers a salinity tolerant phenotype. Furthermore, the GABA and H_2_O_2_ levels are higher and lower, respectively, than the corresponding levels in the *SlGAD1*-4 and *SlGABA-T1-3* knockdown lines (12.5 and 0.5 μmol g^−1^ FM, respectively, vs. 6 and 0.75–0.8 μmol g^−1^ FM). Thus, the tolerance is inversely related with the H_2_O_2_ level.

Overall, these findings suggest that the capacity to tolerate stress is associated with H^+^ consumption during GABA synthesis, pH regulation of H^+^-ATPase, activation of the GABA shunt and TCAC, down-regulation of NADPH oxidase, and GABA inhibition of GORK-mediated K^+^ efflux ([21,60,61,118]; also see Section 3.1). Notably, GABA is implicated in activating the transcription of 14-3-3 proteins, which are known activators of H^+^-ATPases and GORK [119,120].

## 3. GABA Signaling and Its Response to Abiotic Stress

### 3.1. Root Malate and K^+^ Efflux

Wheat root TaALMT1 is responsible for malate efflux, but this is inhibited by GABA binding to the cytosolic surface of the protein [18,101,102] (Figure 1). Indeed, both malate and GABA likely traverse the protein, though not simultaneously or through the same pore [18]. The GABA-binding domain is conserved in the GORK channels of the root epidermis [20]. The *atgork1* mutant displays an hypoxia (waterlogging)-tolerant phenotype, greater K^+^ retention and hypoxia-induced Ca^2+^ signaling, and does not show any change in K^+^ efflux in response to GABA [20,117] (Table 2). Therefore, one would expect the stress-induced accumulation of GABA to reduce the export of malate and K^+^ from root cells [13,18,19,102]. Notably, the GABA-binding domain is conserved in most ALMTs [14] and further research is required to establish if they transport GABA, as well as anions.

### 3.2. Stomata Functioning

ALMTs and GORK facilitate the functioning of stomata [121] (Figure 1). During the opening, malate and Cl- fluxes into the guard cell vacuole are mediated by tonoplast AtALMT6 and malate-activated AtALMT9, respectively. During the closure, the efflux of malate and K^+^ is mediated by plasma membrane-localized AtALMT12 and AtGORK, respectively. The drought-induced increase in abscisic acid (ABA) level upregulates GORK activity, resulting in stomatal closure and drought tolerance. The opening of AtALMT6 and AtALMT4 mediates malate efflux from the vacuole. Various Ca^2+^-permeable channels contribute to the elevation of cytosolic Ca^2+^ during stress [121,122].

A role for GABA accumulation in drought resistance has been shown using *Arabidopsis* mutants with altered GABA levels. The *atgad1/2* double mutant has impaired stomatal closure and a drought-susceptible phenotype [69] (Table 1). Furthermore, the light-to-dark transition is slower, and GABA levels are lower than those in the WT without or with drought. Subsequent research with *atgad2* and *atalmt9* mutants demonstrated that the drought-induced accumulation of GABA suppresses light-induced stomatal opening, whereas it has no effect under constant light [40] (Table 1 and Table 2). Stomatal opening in the *almt12* mutant showed WT sensitivity to GABA, whereas dark-induced stomatal closing is insensitive to GABA [40] (Table 2). These findings indicated that GABA accumulation in the cytosol of the guard cell reduces stomatal reopening and transpirational water loss, thereby improving drought tolerance. Further research is required to investigate iGABA inhibition of ALMT4,6 by cytosolic GABA contributes to the regulation of stomatal movement [123] (Table 2).

### 3.3. Overexpression of Malate Efflux Is Linked to Aluminum Tolerance

The accumulation of free aluminum (Al^3+^) ions in the soil solution under low pH limits plant growth and productivity [124]. Ramesh et al. [101] have monitored malate efflux in roots of near-isogenic, Al^3+^ tolerant (ET8) and sensitive (ES8) lines of wheat. In the absence of Al^3+^, GABA has no effect on malate efflux under acidic conditions. However, malate efflux increases in response to Al^3+^ treatment, and decreases in response to Al^3+^ and exogenous GABA in ET8, but not in ES8. While the GABA level is higher in ET8 than in ES8 under acidic conditions, Al^3+^ reduces the GABA level in both lines to similar levels. This suggests that high endogenous GABA inhibits the activity of TaALMT1 in the absence of Al^3+^, and that both malate and GABA are exported from the cytosol when TaALMT1 is Al^3+^ activated, resulting in the sequestration of Al^3+^ and modulation of the plant sensitivity to Al^3+^ [102,124]. Table 2 summarizes various examples wherein ALMT1 overexpression enhances the efflux of malate in response to Al^3+^ treatment under acidic conditions [101,125,126,127,128]. Further research is required to establish the precise role of GABA efflux under acidic conditions in the presence of Al^3+^ [102]. It could be related to the alleviation of ammonium toxicity [129].

## 4. GABA Metabolism and Its Response to Biotic Stress

### 4.1. Interaction between Plants and Other Organisms

Recent advances in our knowledge of the interactions between plants and other organisms have been reviewed in detail [16,61,130,131,132]. GABA inevitably accumulates in the host plant in response to bacterial and fungal infection, and infestation by invertebrate pests; however, the mechanism of action for GABA appears to differ. For example, the development of insect larvae and root-knot nematodes is delayed, presumably by disrupting the function of neuromuscular junctions [133,134,135,136,137], whereas in bacterial pathogens, quorum sensing is down-regulated, modulating the hypersensitive response in the host [138,139,140,141,142,143,144]. On the other hand, accumulated GABA may boost host endurance against fungal pathogens by sustaining TCAC activity and reducing oxidative damage [132,145,146,147].

### 4.2. Genetic Manipulation of Endogenous GABA Modifies the Biotic Stress Resistance

Table 3 briefly summarizes examples wherein genetic manipulation of endogenous GABA modifies biotic stress resistance. For example, transgenic tobacco and *Arabidopsis* plants with elevated GABA levels are more resistant to infection by *Agrobacterium* and *Pseudomonas* than WT plants [123,124], as well to predation by insect larvae and root-knot nematode [119,120,121,122]. In contrast, tomato plants with extremely low GABA levels are more susceptible to infection by *Ralstonia* [128]. Together, these findings provide strong support for the role of GABA in plant defense [114,115,116].

Recently, Deng et al. [148] reported that activation of the mitogen-activated protein kinase (MPK3/MPK6) signaling cascade greatly induces GABA biosynthesis in *Arabidopsis* (Table 3). The *gad1/2/4* triple and *gad1/2/4/5* quadruple mutants, in which the GABA levels are extremely low and the Glu and alanine levels are compromised, are more susceptible to both Pst and Pst-avrRpt2. Functional loss of AtMPK3/AtMPK6, their upstream AtMKK4/AtMKK5, or their downstream substrate, WRKY33, suppresses *AtGAD1* and *AtGAD4* expression after Pst-avrRpt2 treatment. These findings lend support for involvement of the MPK3/MPK6 signaling cascades in the induction of GAD and plant immunity against bacterial and fungal pathogens [149].

## 5. Exogenous GABA Improves Tolerance to Abiotic and Biotic Stresses

Table 4 briefly summarizes many examples from the literature wherein tolerance to hypoxia, drought, salinity, chilling, heat, osmotic stress and proton stress, as well as heavy metals (i.e., aluminum, arsenic and chromium) is improved in vegetative organs by the application of exogenous GABA [39,150,151,152,153,154,155,156,157,158,159,160,161,162,163,164,165,166,167,168,169,170,171,172,173,174,175,176,177,178,179,180,181,182,183,184]. The application of exogenous GABA typically increases the level of endogenous GABA, and elicits a diverse range of biochemical, molecular and physiological responses. The activity of the GABA shunt is increased to sustain the TCAC and energy production, though the precise response depends on the organ (shoot vs. roots) and the stress under consideration. Activities of N assimilation (including protein degradation) and PA pathways can also be modulated (also see [185]). Elevated endogenous GABA is also responsible for further increasing the stress-induced levels of non-enzymatic (ascorbic acid, GSH, phenols) and enzymatic (e.g., ascorbate oxidase, superoxide dismutase, ascorbate peroxidase, monodehydroascorbate reductase, glutathione reductase, glutathione peroxidase, glutathione S-transferase, catalase) antioxidants and osmolytes (sugars, organic and amino acids, including proline). These result in lower levels of ROS, malondialdehyde (a product of ROS-mediated peroxidation of membrane polyunsaturated fatty acids and marker for the depletion of antioxidant systems), and protein carbonylation (product of protein peroxidation and a marker of oxidative damage), lower activities of NADPH oxidase, lipoxygenase and polyphenol oxidase, and restoration of ion homeostasis (electrolyte leakage), which is indicative of membrane stability.

There is also evidence for the GABA-induced production of nitric oxide (NO) (Table 4), which could be associated with the enhancement of antioxidant defense, as well as regulation of epigenetic mechanisms and gene transcription [157,174,186]. The stress tolerance could also be related to GABA-induced changes in pathways associated with other phytohormones such as ABA (ABA receptors), ethylene (ACC oxidase, ACC synthase), PAs (arginine decarboxylase, free and conjugated forms, S-adenosylmethionine decarboxylase) and salicylate (Table 4) [60,156,172,175,187], which can regulate metabolic homeostasis and influence the expression of stress factors (miRNAs, transcription factors, heat shock proteins) with known and yet-to-be-determined functions [156,183,188]. It is known that the exogenous application of GABA, ABA and salicylate alleviate the drought-induced damage to membranes and leaf water status in creeping bentgrass by affecting similar metabolic pathways, yet cause differential changes in metabolite accumulation [155]. Additionally, NO and nitrate reductase are jointly needed for salicylate-induced water-stress tolerance in pepper plants [189]. The osmotin protein, which belongs to the PR-5 family of pathogenesis-related proteins, is known to inhibit the activity of defensive cell wall barriers in fungi [190]. Cinnamyl alcohol dehydrogenase is involved in lignin biosynthesis and alkenyl reductase and can detoxify cytotoxic substrates such as aldehydes [155]. Further research is required to investigate the effects of exogenous GABA on alternative respiratory pathways involved in the scavenging, regulation and homeostasis of ROS and NO [92,191], the action of other phytohormones [23,156,187,192,193,194,195,196,197], and post-translational modifications and epigenetic regulation of gene expression [147,198] during stress.

## 6. Concluding Remarks

Plants must endure a wide variety of abiotic and biotic stresses under field conditions. To prevent significant yield losses, many crop improvement programs strive to develop stress-tolerant cultivars. Plants may respond uniquely to different or simultaneous stresses, so breeding tolerance against one stress may be at the expense of tolerance to another. With climate change, plants will likely experience more extreme weather events or multiple stresses, including plant diseases. It would therefore be beneficial to develop a tolerance strategy against multiple stresses.

**Figure 2:**
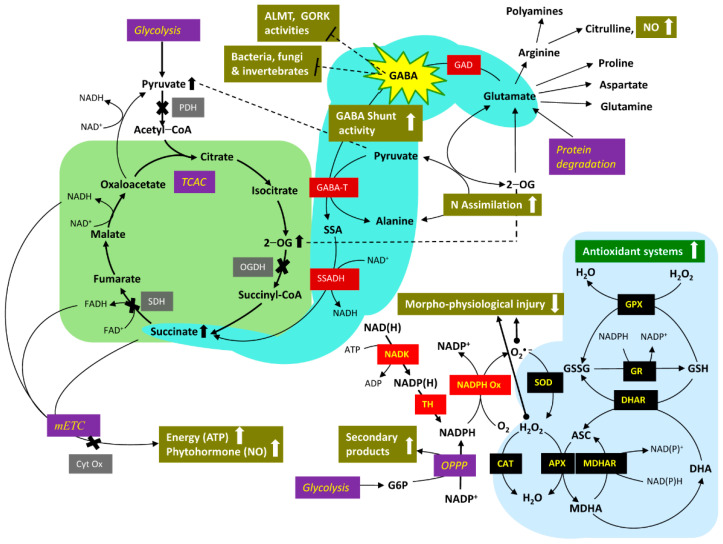
Abiotic and biotic stress-induced changes in morphological and physiological features are restored or partially restored by increasing endogenous GABA and GABA shunt activity. Depending upon the nature of the abiotic stress, the entry of pyruvate into the tricarboxylic acid cycle via pyruvate dehydrogenase, the catabolism of 2−oxoglutarate and succinate via 2−oxoglutarate dehydrogenase and succinate dehydrogenase, respectively, and activity of cytochrome oxidase in the mitochondrial electron transport chain may be restricted, thereby limiting the generation of molecules associated with energy transfer (NADH, FADH and ATP). These limitations are to some extent overcome by increasing the levels of endogenous GABA, by either GAD overexpression or application of exogenous GABA. The elevated endogenous GABA increases flux through the shunt, which in turn, increases the level and/or entry of succinate into the non−cyclic tricarboxylic acid cycle, mitochondrial electron transport chain, and accordingly, ATP generation. The elevated GABA also modifies the activity of stress-induced pathways involving enzymatic and non−enzymatic antioxidants, N assimilation, secondary products, and phytohormones (NO; ethylene and ABA are not shown) by uncharacterized mechanisms. The activity of phytopathogens is directly inhibited by accumulated GABA. Symbols: X, biochemical reaction potentially inhibited by stress; white filled arrows, increasing or decreasing activity of pathway(s) affected by GABA; black filled arrow, increasing level of metabolite affected by stress. Abbreviations: ALMT, aluminum−activated malate transporter; Cyt Ox, cytochrome oxidase; NADK, NAD kinase; OPPP, oxidative pentose phosphate pathway; PDH, pyruvate dehydrogenase; SAM, *S*−adenosylmethionine; SDH, succinate dehydrogenase; TCAC, tricarboxylic acid cycle; TH, transhydrogenase; see Table 4 legend for the remaining abbreviations.

GABA metabolism has garnered considerable attention in recent years, in part because it often accumulates in response to a variety of abiotic (cold, heat, drought, salinity, salinity-alkalinity, osmotic, low O_2_, heavy metal toxicity) and biotic (invertebrate pests, bacteria, fungi) stresses (Figure 2). These findings are typically attributed to stimulation of GABA anabolism or inhibition of GABA catabolism. However, there are clear cases in which GABA pathway activity is promoted to sustain respiration and the generation of energy, without the accumulation of GABA. On the other hand, accumulated GABA may bind to AMLT and GORK, interfering, respectively, with the transport of malate in stomatal guard cells and K^+^ in root epidermal cells, thereby enhancing plant tolerance to drought and hypoxia. With very few exceptions, genetic manipulation of GABA metabolism and receptors, respectively, reveal positive relationships between GABA levels and abiotic/biotic stress tolerance, and between malate efflux from the root and heavy metal tolerance.

Common plant responses to avoid or tolerate abiotic and biotic stresses include stomatal closure and corresponding decreases in photosynthesis, and reduced leaf growth and root length, as well as greater ROS activity. These responses are coordinated by phytohormones such as ABA, NO, ethylene, salicylate and jasmonate. Thus, the enhancement of endogenous GABA by either genetic engineering or the application of exogenous GABA reduces the stress-induced ROS level and restores or partially restores the morpho-physiological features of the unstressed phenotype by promoting or modifying activities of the GABA shunt, TCAC, antioxidant, secondary metabolism and phytohormone pathways (Figure 2). Furthermore, elevated plant GABA adversely affects the activity of phytopathogens by various mechanisms. Therefore, exogenous GABA might function under field conditions as an effective and sustainable tolerance strategy against the multiple abiotic and biotic stresses that could be exacerbated by climate change.

Low temperature and controlled atmosphere conditions (low O_2_, elevated CO_2_) are extensively employed to extend the postharvest life of horticultural commodities, especially botanical fruits. However, these crops may suffer from chilling injury and other physiological disorders, as well as fungal decay. The use of exogenous GABA to improve the marketability of stored horticultural commodities will be described in a companion paper.

## Figures and Tables

**Table 1:** Genetic manipulation of endogenous GABA modifies the abiotic stress phenotype.

Plant Species	Mutant	Abiotic Stress	Biochemical, Molecular and Physiological Responses	References
Arabidopsis (*Arabidopsis thaliana* (L.) Heyhn.)	*pop2-1* *(gaba-t)*	150 mM NaCl for 4 d	↑ GABA (28 μmol g^−1^ DM), Pro (shoot) ↓ DM, Succ, 2-OG, Gln; Glu unaffected (shoot) ↑ GABA (up to 46 μmol g^−1^ DM), Glu, Pro (root) ↓ Gln; Succ and 2-OG unaffected (root)	[68]
	*gad1/2*	100 mM NaCl for 3 wk	↓ FM and DM, Na^+^ uptake (shoot) ↑ GABA by 25% after 7 d (unlike the WT), loss of length ↑ expression of *GORK*, *SKOR* and *RBOHF* (roots) ↑ H_2_O_2_ (0.23 mmol g^−1^ FM), K^+^ efflux (roots) ↓ ATPase activity, MP (roots)	[70]
	*pop2-5* *(gaba-t).*	100 mM NaCl for 3 wk	↓ FM and DM loss, compared to *gad1/2* (leaves) ↑ GABA by 3.5-fold after 7 d (2.1 μmol g^−1^ FM), ATPase activity, MP, *SOS1* and *NHX1* expression more than in *gad1/2* (roots) ↓ H_2_O_2_ level (0.17 mmol g^−1^ FM), *GORK* expression, K^+^ efflux (roots)	[70]
	*aldh10a8,9*	150 mM NaCl for 2 d	↓ GABA from 65 nmol g^−1^ FM in WT to 28–40 nmol g^−1^ FM; Glu unaffected (shoot) ↓ length (roots)	[50]
	*gad1/2*	Drought for 6 d	↓ RWC; delays dark-induced stomatal closure ↑ stomatal conductance, stomatal number; GABA unaffected	[69]
	*gad2*	Drought for 3–7 d	↓ GABA ↑ stomatal conductance, width of stomatal pore; ↓ RWC more quickly *AtGAD2* Ox in guard cells ↑ GABA, ↓ stomatal conductance and ↑ RWC to WT level following 5 d drought	[40]
	*gad1,2*	Waterlogging for 1 wk	↓ shoot FM ↓ GABA (0.8 μmol g^−1^ FM), H_2_O_2_ (0.3 μmol g^−1^ FM) (leaves) ↑ H_2_O_2_ (0.30 μmol g^−1^ FM) within 1 d (roots) ↑ K^+^ efflux (roots), H_2_O_2_-mediated K^+^ efflux, H_2_O_2_-mediated Ca^2+^ influx, OH-mediated K^+^ efflux (roots) ↓ MP, H+ efflux (roots)	[118]
	*gaba-t*	Waterlogging for 1 wk	↑ shoot FM, shoot DM, Chl, Chl fluorescence ↑ GABA (6 μmol g^−1^ FM); ↓ H_2_O_2_ (0.15 μmol g^−1^ FM) (leaves), ↓ H_2_O_2_ (0.15 μmol g^−1^ FM) within 1 d (roots) ↓ K^+^ efflux, H_2_O_2_-mediated K^+^ efflux, H_2_O_2_-mediated Ca^2+^ influx, • OH-mediated K^+^ efflux (roots) ↑ MP, H+ efflux (roots)	[118]
	*gaba-t*	5% O_2_ for 2 d	↑ GABA by 0–15% within 4 h, 15–80% within 48 h in both shoot and roots	[67]
	*ssadh*	37 °C for 2 wk	↑ necrotic lesions, cell death under low fluence white light	[73,74]
Tomato (*Solanum lycopersicum* (L.))	VIGS *GAD1-4*	200 mM NaCl for 3 wk	↓ shoot FM, shoot height ↓ Chl, P_N_ ↑ H_2_O_2_ (0.8 μmol g^−1^ FM) ↓ Glu; GABA (6 μmol g^−1^ FM), Succ, Gln unaffected	[112]
	VIGS *GABA-T1-3*	200 nM NaCl for 3 wk	↓ shoot FM; shoot height unaffected; ↓ Chl, P_N_ ↑ H_2_O_2_ (0.75 μmol g^−1^ FM), GABA (6 μmol g^−1^ FM), Glu ↓ Succ; Gln unaffected	[112]
	VIGS *SSADH*	200 mM NaCl for 4 wk	↑ shoot FM; shoot height unaffected; ↑ Chl, P_N_ ↑ GABA (12.5 μmol g^−1^ FM), Gln ↓ Glu; Succ, H_2_O_2_ unaffected (0.5 μmol g^−1^ FM)	[112]

Symbols: ↑, increases; ↓, decreases. Abbreviations: Chl, chlorophyll; DM/FM, dry/fresh mass; GABA, γ-aminobutyrate; GABA-T, pyruvate/glyoxylate-dependent GABA transaminase; GAD, glutamate decarboxylase; Gln, glutamine; Glu, glutamate; GORK channel, guard cell outward-rectifying K^+^ channel; H_2_O_2_, hydrogen peroxide; MP, membrane potential; NHX and SOS, Na^+^/H^+^ exchanger; O_2_^•−^_,_ superoxide anion; ^•^OH, hydroxyl radical; 2-OG, 2-oxoglutarate; Ox, overexpression; RBOHF, NADPH oxidase; RWC, relative water content; SKOR, stelar K^+^ outward rectifying channel; SSADH, succinic semialdehyde dehydrogenase; Succ, succinate; VIGS, virus-induced gene silencing.

**Table 2:** Genetic manipulation of GABA receptors modifies the abiotic stress phenotype.

Plant Species	Mutant or Transgenic Line	Abiotic Stress	Biochemical, Molecular and Physiological Responses	References
Arabidopsis (*Arabidopsis thaliana* (L.) Heyhn.)	*almt4*	Drought for 2 wk	↑ whole-plant wilting *AtALMT4* Ox complements *atalmt4* phenotype	[123]
	*gork1*	Waterlogging for up to 3 wk	↑ shoot FM, Chl; completes life cycle ↑ K^+^, Ca^2+^ retention in epidermal cells of mature and elongation zones of root ↓ K^+^ efflux rate	[117]
	*almt9*	Drought for up to 7 d	GABA impairs dark-induced stomatal closure *gad2/almt9* retains low GABA of *gad2* and restores stomatal conductance *GAD*∆*C* Ox does not alter stomatal conductance of *almt9* stomatal conductance of *ALMT9^F243C/Y245C^* Ox similar to *gad2* and greater than *almt9*	[40]
	*ZmALMT2* Ox	10 μM Al^3+^ (pH 4.5) for 1 d	↑ length, constitutive and Al^3+^-activated Mal efflux (roots) reverses the Al^3+^-sensitive phenotype of *almt1/mate*	[125]
	*BoALMT* Ox	0.4 mM Al^3+^ (pH 4.5) for 2 d	↑ length, Al^3+^-activated Mal and H+ efflux (roots)	[126]
Arabidopsis and soybean (hairy roots) (*Glycine max* (L.) Merr.)	*GmALMT1* Ox	0.4 mM Al^3+^ (pH 4.5) for 2 d	↑ seedling growth, Mal efflux in root ↓ Al^3+^ content	[127]
Tobacco (*Nicotiana tabaccum* L.)	*MsALMT1* Ox	30 μM Al^3+^ (pH 4.2) for 1 d	↑ root growth ↓ Al^3+^ content	[128]
Barley (*Hordeum vulgare* L.)	*TaALMT1* Ox	0.1 mM Al^3+^ (pH 4.5) for 22 h	↑ root growth in Al^3+^-sensitive line restores Al^3+^-activated, GABA-regulated Mal efflux in root	[101]

Symbols: ↑, increases; ↓, decreases. Abbreviations: Al^3+^, aluminum; ALMT, Al^3+^-activated malate transporter; Chl, chlorophyll; FM, fresh mass; GABA, γ-aminobutyrate; GAD, glutamate decarboxylase; Mal, malate; Ox, overexpression. Species abbreviations: Ms, *Medicago sativa L.* (alfalfa); Bo, *Brassica oleracea* var. *capitata* L. (cabbage); Zm, *Zea mays* L. (corn); Ta, *Triticum aestivum* L. (wheat).

**Table 3:** Genetic manipulation of endogenous GABA modifies the biotic stress phenotype.

Plant Species	Mutant or Transgenic Line	Biotic Stress	Biochemical, Molecular and Physiological Responses	References
Tobacco (*Nicotiana tabaccum* L.)	*GAD*∆*C* Ox	*Meloidogyne hapla* for 9 wk	↓ disease symptoms, nematode egg masses by 50–100%	[42]
		Heliothis virescens	↓ larval feeding by 80–90%	[135]
		*Agrobacterium tumefaciens* for 5 wk	↓ disease symptoms, stem tumor mass by 50% ↑ lactonase *AttM* expression in *Agrobacterium*.	[138]
Arabidopsis (*Arabidopsis thaliana* (L.) Heynh)	*gaba-t*	*Spodoptera littoralis feeding* for 1 wk	↓ larval mass by ~40% ↑ GABA 5-fold after brief feeding	[136,137]
	*gad1/2/4*	*Pseudomonas syringae* pv. *tomato* for 3 d	↓ infection-mediated GABA level ↑ disease incidence induction of *GAD1,4* regulated by *MKK4/MKK5-MPK3/MPK6* cascade and *WRKY33*	[148]
Tobacco or arabidopsis	*atpop* (*gaba-t*), *NtGAD*∆*C* Ox	*Pseudomonas syringae* pv. *tomato* for 2 d	↑ GABA, PR1 expression in plant ↓ expression of bacterial Type III Secretion System with elevated GABA or exogenous GABA	[139,142]
Tomato (*Solanum lycopersicum* L.)	VIGS *GAD2*	*Ralstonia solanacearum* for 4 d	↑ disease incidence	[143]

Symbols: ↑, increases; ↓, decreases. Abbreviations: GABA, γ-aminobutyrate; GABA-T, GABA transaminase; GAD, glutamate decarboxylase; *GAD*∆*C*, GAD lacking the C-terminal calmodulin binding domain; Ox, overexpression; PR, pathogen-related; VIGS, virus-induced gene silencing.

**Table 4:** Exogenous GABA modifies GABA, antioxidant, phytohormone and secondary pathways, and improves tolerance to abiotic stresses.

Plant Species	Abiotic Stress	Biochemical, Molecular and Physiological Responses	References
Arabidopsis (*Arabidopsis thaliana* (L.) Heyhn.)	Drought for up to 7 d	↓ light-stimulated increase in stomatal conductance ↑ WUE	[40]
Tomato (*Solanum lycopersicum* L.)	2 °C for 2 d	↑ GABA; ↓ Pro, soluble sugars, MDA and EL	[150]
	75 mM NaCl for 6 d	↑ growth rate, DM, Chl ↑ GABA, Glu, Pro, activities of GAD, SOD, POD and CAT, expression of *GAD1-3*, especially *GAD1* ↓ ROS, MDA, Na^+^, Na^+^ flux from roots to leaves	[151]
Creeping bentgrass (*Agrostis stolonifera* L.)	35/30 °C or drought for 20–35 d	↑ Chl, RWC, cell membrane stability ↑ Pro ↓ ROS, MDA, EL ↑ expression of *ABF3*, *POD*, *APX*, *HSP90*, *DHN3* and *MT1* during heat ↑ expression of *CDPK26*, *MAPK1*, *ABF3*, *WRKY75*, *MYB13*, *HSP70*, *MT1*, *14-3-3*, *SOD*, *CAT*, *POD*, *APX* and DHAR during drought	[152,153]
	35/30 °C D:N for 35 d	↑ stomatal conductance, WUE, osmotic adjustment ↑ amino acids (GABA), organic acids, sugars, ASC, GSH, GSSG, activities of SOD, APX, DHAR and GR ↓ H_2_O_2_, MDA	[154]
	Drought for 25 d	↑ turf quality, RWC, osmotic adjustment, Chl, photochemical efficiency, amino acids (GABA), organic acids ↓ EL	[155]
	38/33 °C D:N for 12 d	↑ Chl, P_N_ ↑ GABA, expression of *CAD3*, *ACS*, *AER* and many *HSP*s ↓ MDA, EL	[156]
	PEG (−0.52 MPa) for 12 d	↑ RWC, WUE, Chl, P_N_ ↑ GABA, NO, activities of NR, GS, GOGAT, GDH, SOD, CAT, MDHAR and GR ↓ Glu, GAD activity ↓ photoinhibition, ROS, MDA, EL	[157]
Rice (*Oryza sativa* L.)	42/37 °C D:N for 10 d	↑ growth, survival, RWC, stomatal conductance ↑ GABA, Pro, ASC, GSH, activities of CAT and APX ↓ H_2_O, MDA, EL	[158]
	100 μM As^3+^ (pH 4.4) for 22 d	↑ FM, shoot height, P_N_, WUE ↑ GABA, expression of *GAD2,3*, *GABA-T1,2* and *SSADH* (shoot) ↓ activities of CAT, APX, POD, SOD and GR (shoot) ↓ H_2_O_2_, GSH:GSSG (shoot) ↓ expression of *GAD3*, *GABA-T2* and *SSADH* (roots)	[159]
	25 μM As^3+^ (pH 4.4) for 7 d	↑ Pro, Glu, linolenic acid, Put, expression of *ADC* and *SAMDC*, activities of MDHAR, GPX and POD (shoot) ↓ Spd, Spm, ROS (shoot) ↑ length, Put, expression of *ADC* and *SAMDC* (roots) ↓ Spd, Spm, ROS	[160]
	30% PEG or/and 150 mM NaCl or 1 wk	↑ germination percent, vigor index, seedling growth, P_N_, Chl, RWC, WUE ↑ GABA, sugars, protein, starch ↓ Pro, Na^+^, H_2_O_2_, O_2_•−, •OH, MDA ↑ K^+^, activities or/and expression of GR, SOD, CAT, APX, PAL, PPO, SKDH, CAD, GST, CHI, expression of many *CIPK*s,	[161]
White clover (*Trifolium repens* L.)	15% PEG for 17 d	↑ RWC, activities of GABA-T, OGDH, P5CS, PDH, ADC, ODC and SAMDC, *GAD* expression ↑ GABA; transient ↑ Pro ↓ EL, MDA, wilt ↓ Glu, activities of GAD, PAO, CuAO and OAT transient ↑ and ↓ in expression of *ADC*, *ODC*, *SAMDC*, *PAO* and *CuAO* modifies free and conjugated PAs	[162]
	100 mM NaCl for 1 wk	↑ germination percentage, shoot DM and height. ↑ GABA, activities of amylase, CAT, APX, POX, SOD, GR. DHAR and MDHR ↑ expression of *CuZnSOD*, *MnSOD*, *FeSOD*, *CAT*, *APX*, *MDHR*, *GST*, *GPX*, *HKT1*, *HKT8*, *HAL2*, *H+-ATPase*, *SOS1* and genes encoding dehydrins ↓ starch, soluble sugars, amino acids (including Pro) ↓ H_2_O_2_, MDA, EL	[163]
Melon (*Cucumis melo* L. var. *reticulatus*)	2 mg L^−1^ O_2_ for 8 d	↑ GABA, Glu, expression and activities of *ADC*, *ODC* and *SAMDC* (roots) ↓ expression and activities of *DAO* and *PAO* (roots) modifies free and conjugated PAs (roots)	[164]
	50–80 mM Ca(NO3)2 (pH 8.6) for 1 wk	↑ leaf area, P_N_, chloroplast ATPase activities, ALA and improves chloroplast structure (seedling) ↑ GABA, ASC, GSH (seedling) ↑ activities of GAD, GABA-T, PAO, SOD, APX, GR, MDAR and DHAR, expression of *RBOHD* (seedling) ↓ DAO activity, H_2_O_2_, MDA, Chl and its precursors (seedling) ↑ Spd, Spm, activities of ADC, ODC and SAMDC, but ↓ Put (leaves and roots) ↑ GABA-T activity (roots) ↓ activities of GAD, PAO and DAO (roots) Modifies levels of free and conjugated PAs	[165,166,167]
	75 mM NaCl/Na2SO4/NaHCO3/Na2CO3 (1:9:9:1 molar ratio, pH 8.6) for 24 h	↑ plant growth, NO and activities of NR, NOS, SOD, CAT, APX and GR ↓ MDA, restores Na^+^/K^+^ balance	[168]
Mungbean (*Vigna radiata* (L.) R. Wilczek)	45/30 °C D:N for 30 d until maturity	↑ pod number, pod yield, seed yield, P_N_ ↑ sugars, Pro, ASC, GSH ↑ activities of Rubisco, P5CS, PDH, SOD, CAT, APX and GR ↓ H_2_O_2_, MDA	[169]
*Caragana intermedia* Kuang et H.C. Fu	300 mM NaCl for 3 d	↑ GABA; ↓ H_2_O_2_ (roots) transient ↑ in expression of *RBOHB, ACO1,2* and *ABA receptor*, ethylene production (roots) ↓ *CuAO* expression (roots)	[170]
Wheat (*Triticum aestivum* L.)	100–200 mM NaCl for 21 d; 300 mM NaCl for 24 h	↑ germination, shoot DM, Chl, P_N_, activities of SOD and CAT ↓ MDA, EL	[171]
Corn (*Zea mays* L.)	300 mM NaCl for 48 h	↑ shoot height, leaf DM, area per plant, mitochondrial function. ↑ GABA, Pro, sugars, activities of SOD and CAT activities. ↓ GAD activity ↓ MDA	[172]
	waterlogging for 14 d	↑ shoot and root DM, P_N_, Chl, number of grana per chloroplast, GABA, activities of SOD, POD, CAT, GR and APX ↓ MDA, H_2_O_2_, O_2_•−	[173]
Lettuce (*Latuca sativa* L.)	80 mM NaCl for 2 wk	↓ germination time ↑ shoot and root DM, photosynthetic electron flow, activities of CAT, APX and SOD ↓ Pro, H_2_O_2_, EL	[174]
Poplar (*Populus tomentosa* Carr.)	200 mM NaCl for 1 d	↓ H_2_O_2_, C_2_H_4_ (leaves) ↑ ABA (leaves) ↑ H_2_O_2_ (roots) differential expression of various ABA/ethylene biosynthesis and receptor genes	[175]
Perennial ryegrass (*Lolium perenne* L.)	Drought for 14 d	↑ RWC, canopy temperature, turf quality, POD activity ↓ wilt rating, MDA, EL	[176]
Black pepper (*Piper nigrum* L.)	10% PEG (*w*/*v*) for 15 d	↑ RWC, SOD activity ↓ wilting %, Pro, MDA, impact on mitochondrial activity	[177]
Prunus rootstock (*Prunus avium* L.)	4 mg L^−1^ O_2_ for 14 d	transient ↓ and ↑ in H_2_O_2_ in roots and leaves, respectively. ↑ P_N_, stomatal conductance, Chl, Ala (leaves) ↓ Glu (leaves) transient ↑ in Glu, Ala, *GAD2,4* expression (roots)	[178]
Barley (*Hordeum vulgare* L.)	20 μM Al^3+^ (pH 4.5) for 1 d	↑ elongation, activities of SOD, CAT and POD (roots) ↓ protein carbonylation, MDA, ROS	[179]
	20 mM NaCl for 6 d	↑ total phenolics and flavonoids (gallic acid, protocatechic acid, caffeic acid, quercetin), ABTS and DPPH radical scavenging activities ↑ activities or expression of PAL, C4H, 4CL ↓ MDA	[180]
Mustard (*Brassica juncea* L.)	0.15 or 0.3 mM Cr^VI^ for 5 d	↑ shoot height and DM, RWC, Chl, GSH, ASC, GSH/GSSG, ASC/DHA, activities of MDHAR, DHAR, GR, GPX, SOD and CAT ↓ Pro, ROS, MDA, DHA, GSSG, MG, LOX activity	[181]
Tobacco (*Nicotiana tabaccum* L.)	200 μM Cd for 7 d	↑ Pro, GSH, stability of O_2_-evolving complex, electron transfer rate in PSII, stomatal conductance, expression of *P5CS* and *P5CR* ↓ H_2_O_2_, MDA	[182]
Sunflower (*Helianthus annuus* L.)	38 °C or drought 55 d	↑ shoot height and FM, root FM, Chl, transpiration, Pro and TSS ↓ H_2_O_2_, MDA, EL may ↑ activities of SOD and POD, expression of *Hsp70*, *MDHAR*, *Osmotin*, *GR* and *Aquaporin*	[183]
Carrot (*Daucus carota* L.)	Drought for 2 wk	↑ leaf and root growth, RWC, amino acids, CAT activity ↓ MDA, EL	[184]

Symbols: ↑, increases/improves; ↓, decreases. Abbreviations: 14-3-3, regulatory protein; ABA, abscisic acid; ABF, transcription factor; ACS, acetyl-coenzyme A synthetase; AER, NADPH-dependent alkenal reductase P2; As^3+^, arsenic; ASC, ascorbate; ADC, arginine decarboxylase; ALA, δ-aminolevulinic acid; Al^3+^, aluminum; APX, ascorbate peroxidase; CAD3, cinnamyl alcohol dehydrogenase 3; CAT, catalase; Cd, cadmium; CDPK, calcium-dependent protein kinase; Cr^VI^, chromium; CuAO, copper amine oxidase; D, day; DAO, diamine oxidase; DHA, dehydroascorbate; DHAR, dehydroascorbate reductase; DHN, dehydrin; DM/FM, dry/fresh mass; EL, electrolyte leakage; GABA, γ-aminobutyric acid; GABA-TP or GABA-TOG, pyruvate- or 2-oxoglutarate-dependent GABA transaminase; GAD, glutamate decarboxylase; Glu, glutamate; GOGAT, glutamate synthase; GPX, glutathione peroxidase; GR, glutathione reductase; GS, glutamine synthetase; GSH, reduced glutathione; GST, glutathione S-transferase; H_2_O_2_, hydrogen peroxide; HSP, heat shock protein; LOX, lipoxygenase; MAPK, mitogen-activated protein kinase; MDA, malondialdehyde; MDHAR, monodehydroascorbate reductase; miRNA, microRNA; MSI, membrane stability index; MT, metallothionein; MYB, transcription factor; N, night; NO, nitric oxide; NOS, NO synthase; NR, nitrate reductase; OAT, ornithine δ-aminotransferase; ODC, ornithine decarboxylase; P5CR, Δ^1^-pyrroline-5-carboxylate reductase; P5CS, Δ^1^-pyrroline-5-carboxylate synthetase; PA, polyamine; PAO, polyamine oxidase; PEG, polyethyleneglycol 6000; P_N_, net photosynthesis; POD, peroxide dismutase; PPO, polyphenol oxidase; POX, peroxide oxidase; PP, phenylpropanoid; Pro, proline; Put, putrescine; PYL, pyrabactin resistance 1-like; RBOHD, respiratory burst oxidase homologue D/NADPH oxidase; ROS, reactive oxygen species; RuBisCo, ribulose bisphosphate carboxylase oxygenase; RWC, relative water content; SAMDC, S-adenosylmethionine decarboxylase; Spd, spermidine; Spm, spermine; SSADH, succinic semialdehyde dehydrogenase; TSS, total soluble sugars; WRKY, transcription factor; WUE, water use efficiency.

## Supplementary Materials

The following are available online at https://doi.org/10.5281/zenodo.5295721, Table S1: Important *Arabidopsis thaliana* genes associated with GABA metabolism and signaling.

## References

[1] Rockström J., Williams J., Daily G., Noble A., Matthews N., Gordon L., Wetterstrand H., DeClerck F., Shah M., Steduto P. (2017). Sustainable intensification of agriculture for human prosperity and global sustainability. Ambio.

[2] Masson-Delmotte V., Zhai P., Pirani A., Connors S.L., Péan C., Berger S., Caud N., Chen Y., Goldfarb L., Gomis M.I., Intergovernmental Panel on Climate Change (2021). Summary for Policymakers. Climate Change 2021: The Physical Science Basis. Contribution of Working Group I to the Sixth Assessment Report of the Intergovernmental Panel on Climate Change.

[3] Saunders D.G.O. (2021). Will yield gains be lost to disease?. Nat. Clim. Chang..

[4] Godoy F., Olivos-Hernández K., Stange C., Handford M. (2021). Abiotic stress in crop species: Improving tolerance by applying plant metabolites. Plants.

[5] Hasanuzzaman M., Bhuyan M.H.M.B., Zulfiqar F., Raza A., Mohsin S.M., Mahmud J.A., Fujita M., Fotopoulos V. (2020). Reactive oxygen species and antioxidant defense in plants under abiotic stress: Revisiting the crucial role of a universal defense regulator. Antioxidants.

[6] Singh A., Kumar A., Yadav S., Singh I.K. (2019). Reactive oxygen species-mediated signaling during abiotic stress. Plant Gene.

[7] Bown A.W., Shelp B.J. (1997). The metabolism and functions of gamma-aminobutyric acid. Plant Physiol..

[8] Shelp B.J., Bown A.W., McLean M.D. (1999). Metabolism and functions of gamma-aminobutyric acid. Tr. Plant Sci..

[9] Kinnersley A.M., Turano F.J. (2000). Gamma aminobutyric acid (GABA) and plant response to stress. Crit. Rev. Plant Sci..

[10] Bouché N., Lacombe B., Fromm H. (2003). GABA signalling: A conserved and ubiquitous mechanism. Trends Cell Biol..

[11] Bouché N., Fromm H. (2004). GABA in plants: Just a metabolite?. Trends Plant Sci..

[12] Fait A., Fromm H., Walter D., Galili G., Fernie A.R. (2008). Highway or byway: The metabolic role of the GABA shunt in plants. Trends Plant Sci..

[13] Gilliham M., Tyerman S.D. (2016). Linking metabolism to membrane signaling: The GABA–malate connection. Tr. Plant Sci..

[14] Ramesh S.A., Tyerman S.D., Gilliham M., Xu B. (2017). γ-Aminobutyric acid (GABA) signalling in plants. Cell. Mol. Life Sci..

[15] Shelp B.J., Bozzo G.G., Trobacher C.P., Chiu G., Bajwa V.S. (2012). Strategies and tools for studying the metabolism and function of γ-aminobutyrate in plants. I. Pathway structure. Botany.

[16] Shelp B.J., Bown A.W., Zarei A. (2017). 4-Aminobutyrate (GABA): A metabolite and signal with practical significance. Botany.

[17] Shelp B.J., Zarei A. (2017). Subcellular compartmentation of 4-aminobutyrate (GABA) metabolism in arabidopsis: An update. Plant Signal. Behav..

[18] Long Y., Tyerman S.D., Gilliham M. (2020). Cytosolic GABA inhibits anion transport by wheat ALMT1. New Phytol..

[19] Bown A.W., Shelp B.J. (2020). Does the GABA shunt regulate cytosolic GABA?. Tr. Plant Sci..

[20] Adem G.D., Chen G., Shabala L., Chen Z.-H., Shabala S. (2020). GORK channel: A master switch of plant metabolism?. Tr. Plant Sci..

[21] Li L., Dou N., Zhang H., Wu C. (2021). The versatile GABA in plants. Plant Signal. Behav..

[22] Fromm H. (2021). GABA signaling in plants: Targeting the missing pieces of the puzzle. J. Exp. Bot..

[23] Xu B., Sai N., Gilliham M. (2021). The emerging role of GABA as a transport regulator and physiological signal. Plant Physiol..

[24] Wallace W., Secor J., Schraeder L.E. (1984). Rapid accumulation of γ-aminobutyric acid and alanine in soyybean leaves in response to an abrupt transfer to lower temperature, darkness, or mechanical amnipulation. Plant Physiol..

[25] Tuin L.G., Shelp B.J. (1994). In situ [^14^C]glutamate metabolism by developing soybean cotyledons I. Metabolic routes. J. Plant Physiol..

[26] Tuin L.G., Shelp B.J. (1996). In situ [^14^C]glutamate metabolism by developing soybean cotyledons II. The importance of glutamate decarboxylation. J. Plant Physiol..

[27] Breitkreuz K.E., Shelp B.J. (1995). Subcellular compartmentation of the 4-aminobutryate shunt in protoplasts from developing soybean cotyledons. Plant Physiol..

[28] Hijaz F., Killiny N. (2019). Exogenous GABA is quickly metabolized to succinic acid and fed into the plant TCA cycle. Plant Signal. Behav..

[29] Baum G., Lev-Yadun S., Fridman Y., Arazi T., Katsnelson H., Zik M., Fromm H. (1996). Calmodulin binding to glutamate decarboxylase is required for regulation of glutamate and GABA metabolism and normal development in plants. EMBO J..

[30] Ling V., Snedden W.A., Shelp B.J., Assmann S. (1994). Analyses of a soluble calmodulin-binding protein from fava bean roots: Identification as glutamate decarboxylase. Plant Cell.

[31] Arazi T., Baum G., Snedden W.A., Shelp B.J., Fromm H. (1995). Molecular and biochemical analysis of calmodulin interactions with the calmodulin-binding domain of plant glutamate decarboxylase. Plant Physiol..

[32] Snedden W.A., Arazi T., Fromm H., Shelp B.J. (1995). Calcium/calmodulin regulation of soybean glutamate decarboxylase. Plant Physiol..

[33] Snedden W.A., Koutsia N., Baum G., Fromm H. (1996). Activation of a recombinant petunia glutamate decarboxylase by calcium/calmodulin or by a monoclonal antibody which recognizes the calmodulin binding domain. J. Biol. Chem..

[34] Cholewa E., Cholewinski A.J., Shelp B.J., Snedden W.A., Bown A.W. (1997). Cold-shock-stimulated γ-aminobutyric acid synthesis is mediated by an increase in cytosolic Ca^2+^, not by an increase in cytosolic H^+^. Can. J. Bot..

[35] Gut H., Dominici P., Pilati S., Astegno A., Petoukhov M.V., Svergun D.I., Grütter M.G., Capitani G. (2009). A common structural basis for pH- and calmodulin-mediated regulation in plant glutamate decarboxylase. J. Mol. Biol..

[36] Knight H., Trewavas A.J., Knight M.R. (1997). Calcium signaling in *Arabidopsis thaliana* responding to drought and salinity. Plant J..

[37] Bose J., Pottosin I.I., Shabala S.S., Palmgren M.G., Shabala S. (2011). Calcium efflux systems in stress signaling and adaptation in plants. Front. Plant Sci..

[38] Behera S., Xu Z., Luoni L., Bonza M.C., Doccula F.G., De Michelis M.I., Morruis R.J., Schwarzländer M., Costa A. (2018). Cellular Ca^2+^ signals generate defined pH signatures in plants. Plant Cell.

[39] Bouché N., Fait A., Zik A., Fromm H. (2004). The root-specific glutamate decarboxylase (GAD1) for sustaining GABA levels in *Arabidopsis*. Plant Mol. Biol..

[40] Xu B., Long Y., Feng X., Zhu X., Sai N., Chirkova L., Betts A., Herrmann J., Edwards E.J., Okamoto M. (2021). GABA signalling modulates stomatal opening to enhance plant water use efficiency and drought resilence. Nat. Commun..

[41] Zarei A., Chiu G.Z., Yu G., Trobacher C.P., Shelp B.J. (2017). Salinity-regulated expression of genes involved in GABA metabolism and signaling. Botany.

[42] McLean M.D., Yevtushenko A., Deschene A., Van Cauwenberghe O.R., Makhmoudova A., Potter J.W., Bown A.W., Shelp B.J. (2003). Overexpression of glutamate decarboxylase in transgenic tobacco confers resistance to the northern root-knot nematode. Mol. Breed..

[43] Stitti N., Missihoun T., Kotchoni S.S., Kirch H.H., Bartels D. (2011). Aldehyde dehydrogenase in *Arabidopsis thaliana*: Biochemical requirements, metabolic pathways, and functional analysis. Front. Plant Sci..

[44] Shelp B.J., Bozzo G.G., Trobacher C.P., Zarei A., Deyman K.L., Brikis C.J. (2012). Hypothesis/review: Contribution of putrescine to 4-aminobutyrate (GABA) production in response to abiotic stress. Plant Sci..

[45] Moschou P.N., Wu J., Cona A., Tavladoraki P., Angelini R., Roubelakis-Angelakis K.A. (2012). The polyamines and their catabolic products are significant players in the turnover of nitrogenous moleculses in plants. J. Exp. Bot..

[46] Tavladoraki P., Cona A., Angelini R. (2016). Copper-containing amine oxidases and FAD-dependent polyamine oxidases are key players in plant tissue differentiation and organ development. Front. Plant Sci..

[47] Planas-Portell J., Gallart M., Tiburcio A.F., Altabella T. (2013). Copper-containing amine oxidases contribute to terminal polyamine oxidation in peroxisomes and apoplast of *Arabidopsis thaliana*. BMC Plant Biol..

[48] Xing S.G., Jun Y.B., Hau Z.W., Liang L.Y. (2007). Higher accumulation of gamma-aminobutyric acid induced by salt stress through stimulating the activity of diamine oxidases in *Glycine max* (L.) Merr. roots. Plant Physiol. Biochem..

[49] Missihoun T.D., Schmitz J., Klug R., Kirch H.H., Bartels D. (2011). Betaine aldehyde dehydrogenase genes from *Arabidopsis* with different sub-cellular localization affect stress responses. Planta.

[50] Zarei A., Trobacher C.P., Shelp B.J. (2016). *Arabidopsis* aldehyde dehydrogenase 10 family members confer salt tolerance through putrescine-derived 4-aminobutyrate (GABA) production. Sci. Rep..

[51] Jacques F., Zhao Y., Kopečná M., Končitíková R., Kopečný D., Rippa S., Perrin Y. (2020). Roles for ALDH10 enzymes in γ-butyrobetaine synthesis, seed development, germination, and salt tolerance in *Arabidopsis*. J. Exp. Bot..

[52] Signorelli S., Dans P.D., Coitiño E.L., Borsani O., Monza J. (2015). Connecting proline and γ-aminobutyric acid in stressed plants through non-enzymatic reactions. PLoS ONE.

[53] Forlani G., Trovato M., Funck D., Signorelli S., Hossain M.A., Kumar V., Burritt D.J., Fujita M., Mäkelä P.S.A. (2019). Regulation of proline accumulation and its molecular and physiological functions in stress defence. Osmoprotectant-Mediated Abiotic Stress Tolerance in Plants.

[54] Michaeli S., Fait A., Lagor K., Nunes-Nesi A., Grillich N., Yellin A., Bar D., Khan M., Fernie A.R., Turano F.J. (2011). A mitochondrial GABA permease connects the GABA shunt and the TCA cycle, and is essential for normal carbon metabolism. Plant J..

[55] Dündar E., Bush D.R. (2009). BAT1, a bidirectional amino acid transporter in *Arabidopsis*. Planta.

[56] Ariyaratne M., Ge L., Morris P.F. (2019). Characterization of membrane transporters by heterologous expression in *E. coli* and production of membrane vesicles. J. Vis. Exp..

[57] Van Cauwenberghe O.R., Shelp B.J. (1999). Biochemical characterization of partially purified GABA: Pyruvate transaminase from *Nicotiana tabacum*. Phytochemistry.

[58] Clark S.M., Di Leo R., Dhanoa P.K., Van Cauwenberghe O.R., Mullen R.T., Shelp B.J. (2009). Biochemical characterization, mitochondrial localization, expression, and potential functions for an *Arabidopsis* γ-aminobutyrate transaminase that utilizes both pyruvate and glyoxylate. J. Exp. Bot..

[59] Shimajiri Y., Ozaki K., Kainou K., Akama K. (2013). Differential subcellular localization, enzymatic properties and expression patterns of γ-aminobutyric transaminases (GABA-T) in rice (*Oryza sativa*). J. Plant Physiol..

[60] Podlešáková K., Ugena L., Spichal L., Dolezal K., De Diego N. (2019). Phytohormones and polyamines regulate plant stress responses by altering GABA pathway. Nat. Biotechnol..

[61] Seifikalhor M., Aliniaeifard S., Hassani B., Niknam V., Lastochkina O. (2019). Diverse role of γ-aminobutyric acid in dynamic plant cell responses. Plant Cell Rep..

[62] Bandehagh A., Taylor N.L. (2020). Can alternative metabolic pathways and shunts overcome salinity induced inhibition of central carbon metabolism in crops?. Front. Plant Sci..

[63] Babu G.G., Naik G.R. (2013). GABA: Pyruvate-dependent transaminase dominates GABA: 2-oxoglutarate dependent transaminase in sugarcane and their molecular characterization. Int. J. Dev. Res..

[64] Shelp B.J., Walton C.S., Snedden W.A., Tuin L.J., Oresnik I.J., Layzell D.B. (1995). GABA shunt in developing soybean seeds is associated with hypoxia. Physiol. Plant..

[65] Clark S.M., Di Leo R., Van Cauwenberghe O.R., Mullen R.T., Shelp B.J. (2009). Subcellular localization and expression of multiple tomato γ-aminobutyrate transaminases that utilize both pyruvate and glyoxylate. J. Exp. Bot..

[66] Koike S., Matsukura C., Takayama M., Asamizu E., Ezura H. (2013). Suppression of γ-aminobutyric acid (GABA) transaminases induces prominent GABA accumulation, dwarfism and infertility in the tomato (*Solanum lycopersicum* L.). Plant Cell Physiol..

[67] Miyashita Y., Good A.G. (2008). Contribution of the GABA shunt to hypoxia-induced alanine accumulation in roots of *Arabidopsis thaliana*. Plant Cell Physiol..

[68] Renault H., Roussel V., El Amrani A., Arzel M., Renault D., Bouchereau A., Deleu C. (2010). The *Arabidopsis pop2–1* mutant reveals the involvement of GABA transaminase in salt stress tolerance. BMC Plant Biol..

[69] Mekonnen D.W., Flügge U.-I., Ludewig F. (2016). Gamma-aminobutyric acid depletion affects stomata closure and drought tolerance of *Arabidopsis thaliana*. Plant Sci..

[70] Su N., Wu Q., Chen J., Shabala L., Mithöfer A., Wang H., Qu M., Yu M., Cui J., Shabala S. (2019). GABA operates upstream of H^+^-ATPase and improves salinity tolerance in *Arabidopsis* by enabling cytosolic K^+^ retention and Na^+^ exclusion. J. Exp. Bot..

[71] Busch K.B., Fromm H. (1999). Plant succinic semialdehyde dehydrogenase. Cloning, purification, localization in mitochondria, and regulation by adenine nucleotides. Plant Physiol..

[72] Busch K., Piehler J., Fromm H. (2000). Plant succinic semialdehyde dehydrogenase: Dissection of nucleotide binding by surface plasmon resonance and fluorescence spectroscopy. Biochemistry.

[73] Bouché N., Fait A., Bouchez D., Møller S.G., Fromm H. (2003). Mitochondrial succinic-semialdehyde dehydrogenase of the γ-aminobutyrate shunt is required to restrict levels of reactive oxygen intermediates in plants. Proc. Natl. Acad. Sci. USA.

[74] Fait A., Yellin A., Fromm H. (2005). GABA shunt deficiencies and accumulation of reactive oxygen intermediates: Insight from *Arabidopsis* mutants. FEBS Lett..

[75] Jin W., Wu F. (2016). Proteome-wide identification of lysine succinylation in the proteins of tomato (*Solanum lycopsicum*). PLoS ONE.

[76] Zhang K., Xiong Y., Sun W., Wang G.-L., Liu W. (2019). Global proteomic analysis reveals widespread lysine succinylation in rice seedlings. Int. J. Mol. Sci..

[77] Breitkreuz K.E., Allan W.L., Van Cauwenberghe O.R., Jakobs C., Talibi D., André B., Shelp B.J. (2003). A novel γ-hydroxybutyrate dehydrogenase. Identification and expression of an *Arabidopsis* cDNA and potential role under oxygen deficiency. J. Biol. Chem..

[78] Hoover G.J., Van Cauwenberghe O.R., Breitkreuz K.E., Clark S.M., Merrill A.R., Shelp B.J. (2007). Characteristics of an *Arabidopsis* glyoxylate reductase: General biochemical properties and substrate specificity for the recombinant protein, and developmental expression and implications for glyoxylate and succinic semialdehyde metabolism *in planta*. Can. J. Bot..

[79] Simpson J.P., Di Leo R., Allan W.L., Clark S.M., Dhanoa P.K., Makhmoudova A., Hoover G.J., Mullen R.T., Shelp B.J. (2008). Identification and characterization of a plastid-localized *Arabidopsis* glyoxylate reductase isoform: Comparison with a cytosolic isoform and implications for cellular redox homeostasis and aldehyde detoxification. J. Exp. Bot..

[80] Allan W.L., Simpson J.P., Clark S.M., Shelp B.J. (2008). γ-Hydroxybutyrate accumulation in Arabidopsis and tobacco plants is a general response to abiotic stress: Putative regulation by redox balance and glyoxylate reductase isoforms. J. Exp. Bot..

[81] Ching S.L.K., Gidda S.K., Rochon A., van Cauwenberghe O.R., Shelp B.J., Mullen R.T. (2012). Glyoxylate reductase isoform 1 is localized in the cytosol and not peroxisomes in plant cells. J. Integr. Plant Biol..

[82] Brikis C.J., Zarei A., Trobacher C.P., DeEll J.R., Akama K., Mullen R.T., Bozzo G.G., Shelp B.J. (2017). Ancient plant glyoxylate/succinic semialdehyde reductases: GLYR1s are cytosolic, whereas GLYR2s are localized to both mitochondria and plastids. Front. Plant Sci..

[83] Zarei A., Brikis C.J., Bajwa V.S., Chiu G.Z., Simpson J.P., DeEll J.R., Bozzo G.G., Shelp B.J. (2017). Plant glyoxylate/succinic semialdehyde reductases: Comparative biochemical properties, function during chilling stress, and subcellular localization. Front Plant Sci..

[84] Hoover G.J., Prentice G.A., Merrill A.R., Shelp B.J. (2007). Kinetic mechanism of a recombinant *Arabidopsis* glyoxylate reductase: Studies of initial velocity, dead-end inhibition and product inhibition. Can. J. Bot..

[85] Hoover G.J., Jørgensen R., Rochon A., Bajwa V.S., Merrill A.R., Shelp B.J. (2013). Identification of catalytically important amino acid residues for enzymatic reduction of glyoxylate in plants, Biochim. Biophys. Acta Proteins Proteom..

[86] Allan W.L., Breitkreuz K.E., Waller J.C., Simpson J.P., Hoover G.J., Rochon A., Wolyn D.J., Rentsch D., Snedden W.A., Shelp B.J. (2012). Detoxification of succinate semialdehyde in *Arabidopsis* glyoxylate reductase and NAD kinase mutants subjected to submergence stress. Botany.

[87] Zhang Z., Liang X., Lu L., Xu Z., Huang J., He H., Peng X. (2020). Two glyoxylate reductase isoforms are functionally redundant but required under high photorespiration conditions in rice. BMC Plant Biol..

[88] Allan W.L., Clark S.M., Hoover G.J., Shelp B.J. (2009). Role of glyoxylate reductases during stress: A hypothesis. Biochem. J..

[89] Che-Othman M.H., Jacoby R.P., Millar A.H., Taylor N.L. (2020). Wheat mitochondrial respiration shifts from the tricarboxylic cycle to the GABA shunt under salt stress. New Phytol..

[90] Turano F., Dashner R., Upadhyaya A., Caldwell C.R. (1996). Purification of mitochondrial glutamate dehydrogenase from dark-grown soybean seedlings. Plant Physiol..

[91] Monné M., Daddabbo L., Gagneu D., Obata T., Hielscher B., Palmieri L., Miniero D.V., Fernie A.R., Weber A.P.M., Palmieri F. (2018). Uncoupling proteins 1 and 2 (UCP1 and UCP2) from *Arabidopsis thaliana* are mitochondrial transporters of aspartate, glutamate, and dicarboxylates. J. Biol. Chem..

[92] Popov V.N., Syromyatnikov M.Y., Fernie A.R., Chakraborty S., Gupta K.J., Igamberdiev A.U. (2021). The uncoupling of respiration in plant mitochondria: Keeping reactive oxygen and nitrogen species under control. J. Exp. Bot..

[93] Scott-Taggart C.P., Van Cauwenberghe O.R., McLean M.D., Shelp B.J. (1999). Regulation of γ-aminobutyric acid synthesis in situ by glutamate availability. Physiol. Plant..

[94] Renné P., Dreꞵen U., Hebbeker U., Hille D., Flügge U.I., Westhoff P., Weber A.P.M. (2003). The Arabidopsis mutant *dct* is deficient in the plastidic glutamate/malate translocator DiT2. Plant J..

[95] Breitkreuz K.E., Shelp B.J., Fischer W.N., Schwacke R., Rentsch D. (1999). Identification and characterization of GABA, proline and quaternary ammonium compound transporters from *Arabidopsis thaliana*. FEBS Lett..

[96] Grallath S., Weimar T., Meyer A., Gumy C., Suter-Grotemeyer M., Neuhaus J.M., Rentsch D. (2005). The AtProT family. Compatible solute transporters with similar substrate specificity but differential expression patterns. Plant Physiol..

[97] Schwacke R., Grallath S., Breitkreuz K.E., Stransky E., Stransky H., Frommer W.B., Rentsch D. (1999). LeProT1, a transporter for proline, glycine betaine, and γ-aminobutyric acid in tomato pollen. Plant Cell.

[98] Meyer A., Eskandari S., Grallath S., Rentsch D. (2006). AtGAT1, a high affinity transporter for γ-aminobutyric acid and Arabidopsis thaliana. J. Biol. Chem..

[99] Batushansky A., Kirma M., Grillich N., Pham P.A., Rentsch D., Galili G., Fernie A.R., Fait A. (2015). The transporter GAT1 plays an important role in GABA-mediated carbon-nitrogen interactions in Arabidopsis. Front. Plant Sci..

[100] Chung I., Bown A.W., Shelp B.J. (1992). The production and efflux of 4-aminobutyrate in isolated mesophyll cells. Plant Physiol..

[101] Ramesh S.A., Tyerman S.D., Xu B., Bose J., Kaur S., Conn V., Domingos P., Ullah S., Wege S., Shabala S. (2015). GABA signalling modulates plant growth by directly regulating the activity of plant-specific anion transporters. Nat. Commun..

[102] Ramesh S.A., Kamran M., Sullivan W., Chirkova L., Okamoto M., Degryse F., McLaughlin M., Gilliham M., Tyerman S.D. (2018). Aluminum-activated malate transporters can facilitate GABA transport. Plant Cell.

[103] Snowden C.J., Thomas B., Baxter C.J., Smith J.A.C., Sweetlove L.J. (2015). A tonoplast Glu/Asp/GABA exchanger that affects tomato fruit amino acid composition. Plant J..

[104] Kaplan F., Kopka J., Sung D.Y., Zhao W., Popp M., Porat M., Guy C.L. (2007). Transcript and metabolite profiling during cold acclimation of Arabidopsis reveals an intricate relationship of cold-regulated gene expression with modifications in metabolite content. Plant J..

[105] Espinoza C., Degenkolbe T., Caldana C., Zuther E., Leisse A., Willmitzer L., Hincha D.K., Hannah M.A. (2010). Interaction with diurnal and circadian regulation results in dynamic metabolic and transcriptional changes during cold acclimation in *Arabidopsis*. PLoS ONE.

[106] Brikis C.J., Zarei A., Chiu G.Z., Deyman K.L., Liu J., Trobacher C.P., Hoover G.J., Subedi S., DeEll J.R., Bozzo G.G. (2018). Targeted quantitative profiling of metabolites and gene transcripts associated with 4-aminobutyrate (GABA) in apple fruit stored under multiple abiotic stresses. Hort. Res..

[107] Rhodes D.R., Handa S., Bressan R.A. (1986). Metabolic changes associated with adaptation of plant cells to water stress. Plant Physiol..

[108] Mayer R.R., Cherry J.H., Rhodes D. (1990). Effects of heat shock on amino acid metbolism. Plant Physiol..

[109] Satya Narayan V., Nair P.M. (1990). Metabolism, enzymology and possible roles of 4-aminobutyrate in higher plants. Phytochemistry.

[110] Fontaine J.-X., Terce-Laforgue T., Armengaud P., Clement G., Renou J.-P., Pelletier S., Catterou M., Azzopardi M., Gibon Y., Lea P.J. (2012). Characterization of a NADH-dependent glutamate dehydrogenase mutant of *Arabidopsis* demonstrates the key role of this enzyme in root carbon and nitrogen metabolism. Plant Cell.

[111] Carillo P. (2018). GABA shunt in durum wheat. Front. Plant Sci..

[112] Bao H., Chen X., Lv S., Jiang P., Feng J., Fan P., Nie L., Li X. (2015). Virus-induced gene silencing reveals control of reactive oxygen species accumulation and salt tolerance in tomato by *γ*-aminobutyric acid metabolic pathway. Plant Cell Environ..

[113] António C., Päpke C., Rocha M., Diab H., Limami A.M., Obata T., Fernie A.R., van Dongen J. (2016). Regulation of primary metabolism in response to low oxygen availability as revealed by carbon and nitrogen isotope redistribution. Plant Physiol..

[114] Diab H., Limami A. (2016). Reconfiguration of N metabolism upon hypoxia stress and revcovery: Roles of alanine aminottransferase (AlaAT) and glutamate dehydrogense (GDH). Plants.

[115] Vanlerberghe G.C. (2013). Alternative oxidase: A mitochondrial respiratory pathway to maintain metabolic and signaling homeostasis duirng abiotic and biotic stress in plants. Int. J. Mol. Sci..

[116] Ludewig F., Hu A., Fromm H., Beauclair L., Bouché N. (2008). Mutants of GABA transaminase (POP2) suppress the severe phenotype of *succinic semialdehyde dehydrogenase* (*ssadh*) mutants in Arabidopsis. PLoS ONE.

[117] Wang F., Chen Z.-H., Liu X., Colmer T.D., Shabala L., Salih A., Zhou M., Shabala S. (2017). Revealing the roles of GORK channels and NADPH oxidase in acclimation to hypoxia in Arabidopsis. J. Exp. Bot..

[118] Wu Q., Su N., Huang X., Cui J., Shabala L., Zhou M., Yu M., Shabala S. (2021). Hypoxia-induced increase in GABA content is essential for restoration of membrane potential and preventing ROS-induced disturbance to ion homeostasis. Plant Commun..

[119] Lancien M., Roberts M.R. (2006). Regulation of *Arabidopsis thaliana* 14-3-3 gene expression by γ-aminobutyric acid. Plant Cell Environ..

[120] van Kleeff P.J.M., Gao J., Mol S., Zwart N., Zhang H., Li K.W., de Boer A.H. (2018). The *Arabidopsis* GORK K^+^-channel is phosphorylated by calcium-dependent protein kinase 21 (CPK21), which in turn is activated by 14-3-3 proteins. Plant Physiol. Biochem..

[121] Saito S., Uozumi N. (2019). Guard cell membrane anion transport systems and their regulatory components: An elaborate mechanism controlling stress-induced stomatal closure. Plants.

[122] Demidchik V., Shabala S., Isayenkov S., Cuin T.A., Pottosin I. (2018). Calcium transport across plant membranes: Mechanisms and functions. New Phytol..

[123] Eisenach C., Baetz U., Huck N.V., Zhang J., De Angeli A., Beckers G.J.M., Martinoia E. (2017). ABA-induced stomatal closure involves ALMT4, a phosphorylation-dependent vacuolar anion channel of Arabidopsis. Plant Cell.

[124] Kar D., Pradhan A.A., Datta S. (2021). The role of solute transporters in aluminum toxicity and tolerance. Physiol. Plant..

[125] Ligaba A., Kochian L., Piñeros M. (2012). Maize ZmALMT2 is a root anion transporter that mediates constitutive root malate efflux. Plant Cell Environ..

[126] Zhang L., Wu X.-X., Wang J., Qi C., Wang X., Wang G., Li M., Li X., Guo Y.-D. (2018). BoALMT1, an Al-induced malate transporter in cabbage, enhances aluminum tolerance in *Arabidopsis thaliana*. Front. Plant Sci..

[127] Liang C., Piñeros M.A., Tian J., Yao Z., Sun L., Liu J., Shaff J., Coluccio A., Kochian L.V., Liao H. (2013). Low pH, aluminum, and phosphorus coordinately regulate malate exudation through *GmALMT1* to improve soybean adaptation to acid soils. Plant Physiol..

[128] Chen Q., Wu K.-H., Wang P., Yi J., Li K.-Z., Yu Y.-X., Chen L.-M. (2013). Overexpression of MsALMT1, from the aluminum-sensitive *Medicago sativa*, enhances malate exudation and aluminum resistance in tobacco. Plant Mol. Biol. Rep..

[129] Ma X., Zhu C., Yang N., Gan L., Xia K. (2016). γ-Aminobutyric acid addition alleviates ammonium toxicity by limiting ammonium accumulation in rice (*Oryza sativa*) seedlings. Physiol. Plant..

[130] Bown A.W., MacGregor K.B., Shelp B.J. (2006). Gamma-aminobutyrate: Defence against invertebrate pests?. Trends Plant Sci..

[131] Shelp B.J., Bown A.W., Faure D. (2006). Extracellular γ-aminobutyrate mediates communication between plants and other organisms. Plant Physiol..

[132] Tarkowski K.P., Signorelli S., Höfte M. (2020). γ-Aminobutyric acid and related amino acids in plant immune responses: Emerging mechanisms of action. Plant Cell Environ..

[133] Ramputh A., Bown A.W. (1996). Rapid gamma-aminobutyric acid synthesis and the inhibition of the growth and development of oblique-banded leaf-roller larvae. Plant Physiol..

[134] Bown A.W., Hall D.E., MacGregor K.B. (2002). Insect footsteps on leaves stimulate the accumulation of 4-aminobutyrate and can be visualized through increased chlorophyll fluorescence and superoxide production. Plant Physiol..

[135] MacGregor K.E., Shelp B.J., Peiris S.E., Bown A.W. (2003). Overexpression of glutamate decarboxylase in transgenic tobacco deters feeding by phytophagous insect larvae. J. Chem. Ecol..

[136] Scholz S.S., Reichelt M., Mekonnen D.W., Ludewig F., Mithöfer A. (2015). Insect herbivory-elicited GABA accumulation in plants is a wound-induced, direct, systemic, and jasmonate-independent defense response. Front. Plant Sci..

[137] Scholz S.S., Malabarba J., Reichelt M., Heyer M., Mekonnen D.W., Ludewig F., Mithöfer A. (2017). Evidence for GABA-induced systemic accumulation in Arabidopsis upon wounding. Front. Plant Sci..

[138] Chevrot R., Rosen R., Haudecoeur E., Cirou A., Shelp B.J., Ron E., Faure D. (2006). GABA controls the level of quorum-sensing signal in *Agrobacterium tumefaciens*. Proc. Natl. Acad. Sci. USA.

[139] Park D.H., Mirabella R., Bronstein P.A., Preston G.M., Haring M.A., Lim C.K., Collmer A., Schuurink R.C. (2010). Mutations in γ-aminobutyric acid (GABA) transaminase genes in plants or *Pseudomonas syringae* reduce bacterial virulence. Plant J..

[140] Planamente S., Mondy S., Hommais F., Vigouroux A., Moréra S., Faure D. (2012). Structural basis for selective GABA binding in bacterial pathogens. Mol. Microbiol..

[141] Kim N.H., Kim B.S., Hwang B.K. (2013). Pepper arginine decarboxylase is required for polyamine and γ-aminobutyric acid signaling in cell death and defense response. Plant Physiol..

[142] McCraw S.L., Park D.H., Jones R., Bentley M.A., Rico A., Ratcliffe R.G., Kruger N.J., Collmer A., Preston G.M. (2016). GABA (γ-aminobutyric acid) uptake via the GABA permease GabP represses virulence gene expression in *Pseudomonas syringae* pv. tomato DC3000. Mol. Plant-Microbe Interact..

[143] Wang G., Kong J., Cui D., Zhao H., Niu Y., Xu M., Jiang G., Zhao Y., Wang W. (2019). Resistance against *Ralstonia solanacearum* in tomato depends on the methionine cycle and the γ-aminobutyric acid metabolic pathway. Plant J..

[144] Seifi H.S., Curvers K., De Vleesschauwer D., Delaere I., Aziz A., Hofte M. (2013). Concurrent overactivation of the cytosolic glutamine synthetase and the GABA shunt in the ABA-deficient sitiens mutant of tomato leads to resistance against *Botrytis cinerea*. New Phytol..

[145] Gupta K., Sengupta A., Chakraborty M., Gupta B. (2016). Hydrogen peroxide and polyamines act as double edged swords in plant abiotic stress responses. Front. Plant Sci..

[146] Seifi H.S., Shelp B.J. (2019). Spermine differentially refines plant defense responses against biotic and abiotic stresses. Front. Plant Sci..

[147] Seifi H.S., Zarei A., Hsiang T., Shelp B.J. (2019). Spermine is a potent plant defense activator against gray mold disease on *Solanum lycopersicum*, *Phaseolus vulgaris*, and *Arabidopsis thaliana*. Phytopathology.

[148] Deng X., Xu X., Liu Y., Zhang Y., Yang L., Zhang S., Xu J. (2020). Induction of γ-aminobutyric acid plays a positive role to *Arabidopsis* resistance against *Pseudomonas syringae*. J. Integr. Plant Biol..

[149] Asano T., Nguyen T.H.-N., Yasuda M., Sidiq Y., Nishimura K., Nakashita H., Nishiuchi T. (2020). Arabidopsis MAPKKK **δ**-1 is required for full immunity against bacterial and fungal infection. J. Exp. Bot..

[150] Malekzadeh P., Khara J., Heydari R. (2014). Alleviating effects of exogenous gamma-aminobutiric acid on tomato seedling under chilling stress. Physiol. Mol. Biol. Plants.

[151] Wu X., Jia Q., Ji S., Gong B., Li J., Lü G., Gao H. (2020). Gamma-aminobutyric acid (GABA) alleviates salt damage in tomato by modulating Na^+^ uptake, the *GAD* gene, amino acid synthesis and reactive oxygen species metabolism. BMC Plant Biol..

[152] Li Z., Peng Y., Huang B. (2016). Physiological effects of γ-aminobutyric acid application on improving heat and drought tolerance in creeping bentgrass. J. Am. Soc. Hort. Sci..

[153] Li Z., Peng Y., Huang B. (2018). Alteration of transcripts of stress-protective genes and transcriptional factors by γ-aminobutyric acid (GABA) associated with improved heat and drought tolerance in creeping bentgrass (*Agrostis stolonifera*). Int. J. Mol. Sci..

[154] Li Z., Yu J., Peng Y., Huang B. (2016). Metabolic pathways regulated by γ-aminobutyric acid (GABA) contributing to heat tolerance in creeping bentgrass (*Agrostis stolonifera*). Sci. Rep..

[155] Li Z., Yu J., Peng Y., Huang B. (2017). Metabolic pathways regulated by abscisic acid, salicylic acid and γ-aminobutyric acid in association with improved drought tolerance in creeping bentgrass (*Agrostis stolonifera*). Physiol. Plant..

[156] Li Z., Cheng B., Zeng W., Liu Z., Peng Y. (2019). The transcriptional and post-transcriptional regulation in perennial creeping bentgrass in response to γ-aminobutyric acid (GABA) and heat stress. Environ. Exp. Bot..

[157] Tang M., Li Z., Luo L., Cheng B., Zhang Y., Zeng W., Peng Y. (2020). Nitric oxide signal, nitrogen metabolism, and water balance affected by γ-aminobutyric acid (GABA) in relation to enhanced tolerance to water stress in creeping bentgrass. Int. J. Mol. Sci..

[158] Nayyar H., Kaur H., Kaur S., Singh R. (2014). γ-Aminobutyric acid (GABA) imparts partial protection from heat stress injury to rice seedlings by improving leaf turgor and upregulating osmoprotectants and antioxidants. J. Plant Growth Regul..

[159] Kumar N., Dubey A.K., Upadhyay A.K., Gautam A., Ranjan R., Srikishna S., Sahu N., Behera S.K., Mallick S. (2017). GABA accretion reduces *Lsi-1* and *Lsi-2* gene expressions and modulates physiological responses in *Oryza sativa* to provide tolerance towards arsenic. Sci. Rep..

[160] Kumar N., Gautam A., Dubey A.K., Ranjan R., Pandey A., Kumari B., Singh G., Mandotra S., Chauhan P.S., Srikrishna S. (2019). GABA mediated reduction of arsenite toxicity in rice seedling through modulation of fatty acids, stress responsive amino acids and polyamines biosynthesis. Ecotoxicol. Environ. Saf..

[161] Sheteiwy M.S., Shao H., Qi W., Hamoud Y.A., Shaghaleh H., Khan N.U., Yang R., Tang B. (2019). GABA-alleviated oxidative injury induced by salinity, osmotic stress and their combination by regulating cellular and molecular signals in rice. Int. J. Mol. Sci..

[162] Yong B., Xie H., Li Z., Li Y.-P., Zhang Y., Nie G., Zhang X.-Q., Ma X., Huang L.-K., Yan Y.-H. (2017). Exogenous application of GABA improves PEG-induced drought tolerance positively associated with GABA-shunt, polyamines, and proline metabolism in white clover. Front. Physiol..

[163] Cheng B., Li Z., Liang L., Cao Y., Zeng W., Zhang X., Ma X., Huang L., Nie G., Liu W. (2018). The γ-aminobutyric acid (GABA) alleviates salt stress damage during seeds germination of white clover associated with Na^+^/K^+^ transportation, dehydrins accumulation, and stress-related genes expression in white clover. Int. J. Mol. Sci..

[164] Wang I., Fan L., Gao H., Wu X., Li J., Lv G., Gong B. (2014). Polyamine biosynthesis and degradation are modulated by exogenous gamma-aminobutyric acid in root-zone hypoxia-stressed melon roots. Plant Physiol. Biochem..

[165] Hu X., Xu Z., Xu W., Li J., Zhao N., Zhou Y. (2015). Application of γ-aminobutyric acid demonstrates a protective role of polyamine and GABA metabolism in muskmelon seedlings under Ca(NO_3_)_2_ stress. Plant Physiol. Biochem..

[166] Xiang L., Hu L., Xu W., Zhen A., Zhang L., Hu X. (2016). Exogenous γ-aminobutyric acid improves the structure and function of photosystem II in muskmelon seedlings exposed to salinity-alkalinity stress. PLoS ONE.

[167] Jin X., Liu T., Xu J., Gao Z., Hu X. (2019). Exogenous GABA enhances muskmelon tolerance to salinity-alkalinity stress by regulating redox balance and chlorophyll biosynthesis. BMC Plant Biol..

[168] Xu J., Liu T., Qu F., Jin X., Huang N., Wang J., Hu X. (2021). Nitric oxide mediates γ-aminobutyric acid-enhanced muskmelon tolerance to salinity–alkalinity stress conditions. Sci. Hortic..

[169] Priya M., Sharma L., Kaur R., Bindumadhava H., Nair R.M., Siddique K.H.M., Nayyar H. (2019). GABA (γ-aminobutyric acid), as a thermo-protectant, to improve the reproductive function of heat-stressed mungbean plants. Sci. Rep..

[170] Shi S.-Q., Shi Z., Jiang Z.-P., Qi L.-W., Sun X.-M., Li C.X., Liu J.F., Xiao W.F., Zhang S.-G. (2010). Effects of exogenous GABA on gene expression of *Caragana intermedia* roots under NaCl stress: Regulatory roles for H_2_O_2_ and ethylene production. Plant Cell Environ..

[171] Li M.F., Guo S.J., Yang X.H., Meng Q.W., Wei X.J. (2016). Exogenous gamma-aminobutyric acid increases salt tolerance of wheat by improving photosynthesis and enhancing activities of antioxidant enzymes. Biol. Plant..

[172] Wang Y., Gu W., Meng Y., Xie T., Li L., Li J., Wei S. (2017). γ-Aminobutyric acid imparts partial protection from salt stress injury to maize seedlings by improving photosynthesis and upregulating osmoprotectants and antioxidants. Sci. Rep..

[173] Salah A., Zhan M., Cao C., Han Y., Ling L., Liu Z., Li P., Ye M., Jiang Y. (2019). γ-Aminobutyric acid promotes chloroplast ultrastructure, antioxidant capacity, and growth of waterlogged maize seedlings. Sci. Rep..

[174] Kalhor M.S., Aliniaeifard S., Seif M., Asayesh E.J., Bernard F., Hassani B., Li T. (2018). Enhanced salt tolerance and photosynthetic performance: Implication of ɤ-amino butyric acid application in salt-exposed lettuce (*Lactuca sativa* L.) plants. Plant Physiol. Biochem..

[175] Ji J., Yue J., Xie T., Chen W., Du C., Chang E., Chen L., Jiang Z., Shi S. (2018). Roles of γ-aminobutyric acid on salinity-responsive genes at transcriptomic level in poplar: Involving in abscisic acid and ethylene-signalling pathways. Planta.

[176] Krishnan S., Laskowski K., Shukla V., Merewitz E.B. (2013). Mitigation of drought stress damage by exogenous application of a non-protein amino acid γ–aminobutyric acid on perennial ryegrass. J. Am. Soc. Hort. Sci..

[177] Vijayakumari K., Puthur J.T. (2016). γ-Aminobutyric acid (GABA) priming enhances the osmotic stress tolerance in *Piper nigrum* Linn. plants subjected to PEG-induced stress. Plant Growth Regul..

[178] Salvatierra A., Pimentel P., Almada R., Hinrichsen P. (2016). Exogenous GABA application transiently improves the tolerance to root hypoxia on a sensitive genotype of *Prunus* rootstock. Environ. Exp. Bot..

[179] Song H., Xu X., Wang H., Wang H., Tao Y. (2010). Exogenous *γ*-aminobutyric acid alleviates oxidative damage caused by aluminium and proton stresses on barley seedlings. J. Sci. Food Agric..

[180] Wang M., Zhu Y., Wang P., Gu Z., Yang R. (2021). Effect of γ-aminobutyric acid on phenolics metabolism in barley seedlings under low NaCl treatment. Antioxidants.

[181] Mahmud J.A.L., Hasanuzzaman M., Nahar K., Rahman A., Hossain M.S., Fujita M. (2017). γ-Aminobutyric acid (GABA) confers chromium stress tolerance in *Brassica juncea* L. by modulating the antioxidant defense and glyoxalase systems. Ecotoxicology.

[182] He G., Zhang H., Liu S., Li H., Huo Y., Guoc K., Xu Z., Zhang H. (2021). Exogenous γ-glutamic acid (GABA) induces proline and glutathione synthesis in alleviating Cd-induced photosynthetic inhibition and oxidative damage in tobacco leaves. J. Plant Interact..

[183] Razik E.S.A., Alharbi B.M., Pirzadah T.B., Alnusairi G.S.H., Soliman M.H., Hakeem K.R. (2021). γ-Aminobutyric acid (GABA) mitigates drought and heat stress in sunflower (*Helianthus annuus* L.) by regulating its physiological, biochemical and molecular pathways. Physiol. Plant..

[184] Bashir R., Riaz H.N., Anwar S., Parveen N., Khalilzadeh R., Hussain I., Mahmood S. (2021). Morpho-physiological changes in carrots by foliar γ-aminobutyric acid under drought stress. Braz. J. Bot..

[185] Chen D., Shao Q., Yin L., Younis A., Zheng B. (2019). Polyamine function in plants: Metabolism, regulation on development, and roles in abiotic stress responses. Front. Plant Sci..

[186] Ageeva-Kieferle A., Georgii E., Winkler B., Ghirardo A., Albert A., Hüther P., Mengel A., Becker C., Schnitzler J.-P., Durner J. (2021). Nitric oxide coordinates growth, development, and stress response via histone modification and gene expression. Plant Physiol..

[187] Hijaz H., Nehela Y., Killiny N. (2018). Application of gamma-aminobutyric acid increased the level of phytohormones in *Citrus sinensis*. Planta.

[188] Renault H., El Amrani A., Palanivelu R., Updegraff E.P., Yu A., Renou J.-P., Preuss D., Bouchereau A., Deleu C. (2011). GABA accumulation causes cell elongation defects and a decrease in expression of genes encoding secreted and cell wall-related proteins in *Arabidopsis thaliana*. Plant Cell Physiol..

[189] Kaya C. (2021). Nitrate reductase is required for salicylic acid-induced water stress tolerance of pepper by upraising the AsA-GSH pathway and glyoxalase system. Physiol. Plant..

[190] Ullah A., Hussain A., Shaban M., Khan A.M., Alariqi M., Gul M., Jun Z., Lin S., Li J., Jin S. (2018). Osmotin: A plant defense tool against biotic and abiotic stresses. Plant Physiol. Biochem..

[191] Patel M.K., Pandey S., Burritt D.J., Tran L.-S.P. (2019). Plant responses to low-oxygen stress: Interplay between ROS and NO signaling pathways. Environ. Exp. Bot..

[192] Liu T., Xu T., Li J., Hu X. (2019). NO is involved in JA- and H_2_O_2_-mediated ALA-induced oxidative stress tolerance at low temperatures in tomato. Environ. Exp. Bot..

[193] Prakash V., Singh V.P., Tripathi D.K., Sharma S., Corpas F.J. (2019). Crosstalk between nitric oxide (NO) and abscisic acid (ABA) signalling molecules in higher plants. Environ. Exp. Bot..

[194] Bharath P., Gahir S., Raghavendra A.S. (2021). Abscisic acid-induced stomatal closure: An important component of plant defense against abiotic and biotic stress. Front. Plant Sci..

[195] Corpas F.J., González-Gordo S., Palma J.M. (2021). Nitric oxide and hydrogen sulfide modulate the NADPH generating enzymatic system in higher plants. J. Exp. Bot..

[196] Cui B., Xu S., Li Y., Umbreen S., Frederickson D., Yuan B., Jiang J., Liu F., Pan Q., Loake G.J. (2021). The Arabidopsis zinc finger proteins SRG2 and SRG3 are positive regulators of plant immunity and are differentially regulated by nitric oxide. New Phytol..

[197] Manrique-Gil I., Sánchez-Vicente I., Torres-Quezada I., Lorenzo O. (2021). Nitric oxide function during oxygen deprivation in physiological and stress processes. J. Exp. Bot..

[198] Wurm C.J., Lindermayr C. (2021). Nitric oxide signaling in the plant nucleus: The function of nitric oxide in chromatin modulation and transcription. J. Exp. Bot..

